# Assessment of Convolutional Neural Network and Vision Transformer Architectures for Structural Surface Classification

**DOI:** 10.3390/jimaging12100487

**Published:** 2026-10-06

**Authors:** Bonginkosi A. Thango, Sipho G. Thango

**Affiliations:** 1 Department of Electrical and Electronic Engineering Technology, University of Johannesburg, Johannesburg 2092, South Africa; 2 Discipline of Civil Engineering, University of KwaZulu Natal, Durban 4041, South Africa; thangos@ukzn.ac.za or siphot@nhbrc.org.za; 3 National Home Builders Registration Council, Durban 4001, South Africa

**Keywords:** structural surface classification, StructDamage, convolutional neural network, transformer/attention, transfer learning, related-image leakage, training-budget sensitivity, reproducibility

## Abstract

We evaluate ten convolutional neural networks (CNNs) and ten transformer/attention architectures for nine-category surface classification using 41,756 balanced StructDamage images. A frozen ImageNet-pretrained ResNet18 encoder and learned head use 33,352 training, 4202 validation and 4202 test images grouped by inferred augmentation family. Test accuracy is 98.88%, macro-F1 is 0.9881, and the 95% component-bootstrap accuracy interval is 98.42–99.26%. Content screening retains 4102 test images and 98.85% accuracy. Nevertheless, cross-partition exact matches and candidate source-prefix overlap prevent treating these results as independent-site validation. The separate random-initialization screen uses 1800 training images, 900 validation images, the common test set, 64 × 64 inputs and five epochs. DenseNet121 leads with 88.34% accuracy; XCiT-Tiny leads the transformer/attention group with 84.75% accuracy. Eighteen models select the final epoch, so the comparison does not establish convergence-equivalent superiority. With 5400 training images, all twenty accuracies increase and the between-budget rank correlation is 0.850. Component-aware comparisons and wall/deck error analysis qualify the ranking. The findings concern surface-category recognition, not defect localization, damage severity or structural safety.

## 1. Introduction

Image-based inspection provides a way to organize and examine visible surface information from buildings, pavements and other civil infrastructure. Its usefulness depends on matching the model output to the engineering question: identifying a surface, detecting a visible defect and assessing structural safety are different tasks. Convolutional neural networks (CNNs) have enabled learned representations for pavement crack recognition [1] and concrete crack detection [2], reducing reliance on manually designed image descriptors. These developments motivate the automated analysis of inspection imagery, but an image-classification result alone does not establish the physical cause, severity or consequences of deterioration. Progress in this area has involved both data resources and prediction methods. Annotated collections such as SDNET2018 support reusable evaluation [3], while hierarchical convolutional features [4], contextual attention [5] and transformer-based crack models [6] address increasingly detailed visual patterns. Boundary-aware hybrid networks [7], transformer-based image classifiers [8] and CNN–transformer segmentation models [9] also show that related structural imagery can support different forms of supervision. Their outputs range from a label for an entire patch to a mask for individual crack pixels. A meaningful comparison must therefore distinguish between these targets before interpreting differences in reported performance. This study focuses specifically on structural surface classification using a local balanced derivative of StructDamage [10]. Each image receives one of nine supplied folder labels: brick, cob, concrete, decks, pavements, road, stone, tile or walls. The labels combine material descriptions with surface or structural-element context; they are not a universally exclusive material taxonomy. Structural damage denotes physical deterioration or impairment of an element, whereas surface condition describes its visible state, including appearance features that do not necessarily signify damage. Here, the supervised output is the supplied surface category. A crack visible in an image labeled ‘brick’ does not convert the prediction into a crack diagnosis, severity grade or safety decision. The practical question is how reliably these categories can be distinguished under limited computational resources, and how that assessment changes when image dependence and training conditions are considered. Augmented variants and related crops can retain shared visual content across partitions. Separately, pretraining, image resolution, sample count and optimization exposure can change a model’s observed ranking. These are distinct issues: strong accuracy within a consolidated collection does not by itself demonstrate performance on an independent structure, and a short training schedule does not establish the performance ceiling of an architecture. This study therefore treats partition provenance, model configuration and uncertainty as part of the interpretation, rather than relying on headline accuracy alone.

The experimental design separates two questions. A full-data pipeline evaluates a frozen pretrained ResNet18 encoder with a learned classifier head on 41,756 images. A separate screen compares ten CNNs and ten transformer/attention architectures initialized randomly under a common five-epoch, low-resolution protocol, followed by an enlarged-training-subset sensitivity analysis. The contributions are an explicitly defined nine-category benchmark; filename-family partitioning combined with content-based dependence screening; saved per-image predictions for twenty named implementations; and component-aware uncertainty, training-history assessment, resource comparisons and class-wise error analysis. The purpose is to support conditional model selection under documented constraints, not to introduce a new architecture or claim an exhaustive, convergence-equivalent comparison.

Section 2 reviews the historical development, recent methods and remaining evaluation gaps. Section 3 describes the dataset, partitioning, model implementations, training and statistical procedures. Section 4 presents the two experimental tiers, dependence audits, training-budget sensitivity and wall/deck error analysis. Section 5 states the conclusions and their limits for engineering use.

## 2. Literature Review

Research on structural-surface imagery has developed along several related but non-equivalent directions: patch-level crack recognition, pixel-level segmentation, surface-category classification and benchmark construction. Table 1 summarizes selected developments from 2016 to 2026, including dataset consolidation, efficient sequence models and synthetic supervision. The following synthesis examines what these studies establish, the conditions under which their results apply, and the evaluation requirements that remain for the present nine-category task.

### 2.1. From Patch Classification to Context-Aware Segmentation

The early CNN studies established that learned features can separate crack-bearing image patches from background appearance. Zhang et al. [1] addressed pavement crack detection, while Cha et al. [2] combined patch classification with sliding-window analysis of larger concrete images. These formulations connect local texture learning to image inspection, but the classification unit remains a patch or window. A positive patch prediction does not provide a precise crack boundary. Cha et al.’s assessment on larger images from a different structure also illustrates why evaluation beyond a random partition of related patches is relevant to the intended inspection setting [2]. DeepCrack shifted the emphasis toward dense prediction by combining hierarchical convolutional features across scales [4]. Shallow spatial detail and deeper contextual information serve complementary roles when the target is a thin, connected defect rather than an entire image category. Xiang et al. [5] subsequently used pyramid context and spatial–channel attention to combine contextual information with local crack detail and suppress irrelevant responses. Attention in structural imagery therefore predates the widespread use of vision transformers. Together, these studies motivate multiscale and contextual representations, but their pixel-level objectives cannot be evaluated using only the nine folder labels available here.

This distinction also affects the interpretation of metrics. Patch accuracy asks whether an image label is correct, whereas segmentation evaluates agreement over labeled pixels. A method that produces continuous crack masks may improve a localization measure without changing a surface-classification score, and a classifier may recognize concrete texture without identifying any crack. Consequently, the methodological connection between these studies and the present benchmark is representational, not an equivalence of targets or reported accuracies [1,2,4,5].

### 2.2. Transformer, Hybrid and Lightweight Sequence Models

CrackFormer introduced transformer-based processing for fine-grained crack detection, using attention to model relationships beyond a local convolutional neighborhood [6]. This is particularly relevant to elongated defects whose visible segments may be spatially separated. Tao et al. [7] combined dilated convolutional features, transformer context and boundary-aware supervision. This hybrid design illustrates that improved crack localization can arise from the interaction between architecture and task-specific training signals, rather than from attention alone. Such supervision is absent when an experiment supplies only one surface-category label per image. Recent work continues to distinguish classification from segmentation. Ye et al. [8] used an improved residual cross-window transformer for binary concrete crack classification, reporting 96.92% accuracy on their own 17,000-image dataset. Ye et al. [9] developed transFissNet, combining ResNet101 features with transformer processing for concrete crack segmentation. These studies support the use of local and broader contextual features, but neither provides a directly paired baseline for the present nine-class screen. Their label definitions, inputs and training settings differ. Importing a reported accuracy as an architecture ranking would confound these experimental differences with the model itself.

The architectural landscape also extends beyond a binary CNN-versus-transformer distinction. Liu et al.’s SCSegamba [13] uses a structure-aware visual state-space module, gated bottleneck convolution and a scanning strategy designed to preserve crack morphology and texture relationships. Its reported 2.8-million-parameter configuration highlights the continuing interest in efficient dense prediction. The relevance here is the need to consider representation and resource use together, not a claim that the twenty evaluated models cover every recent architecture. State-space segmentation models and their specialized supervision are outside of the comparison carried out here.

### 2.3. Dataset Consolidation, Transfer Learning and Synthetic Supervision

Dataset construction has become a substantive research contribution rather than a preliminary step. SDNET2018 provides more than 56,000 annotated sub-images derived from 230 parent photographs of decks, walls and pavements [3]. Its parent-image structure illustrates why the number of stored patches is not necessarily the number of independent observations. CrackSeg9k [11] addresses a complementary problem by combining existing crack datasets, harmonizing annotations and handling source inconsistencies such as noise and distortion. Its benchmark supports comparison under shared data and metrics. Consolidation improves accessibility, but it does not remove the need to track the sources from which images and labels were obtained. Benz and Rodehorst’s Omni-Crack30k [12] broadens this direction through a crack-segmentation benchmark assembled from more than twenty datasets and approximately 30,000 samples. The study evaluates crack-specific methods alongside transfer learning with general-purpose segmentation approaches. This makes the training representation an explicit part of the comparison: a pretrained system and a randomly initialized model do not answer the same resource question. For the present study, it motivates reporting the full-data-pretrained feature pipeline separately from the randomly initialized architecture screen, rather than interpreting their accuracy gap as an isolated architectural effect. StructDamage [10] extends multi-source consolidation to a unified classification collection. The present experiment uses its local 41,756-image balanced derivative and the supplied surface-category folders, not the full public release or a newly annotated damage-severity dataset. Aggregation across sources increases the variety of images available for training, but source appearance can also become associated with category labels. Accordingly, release identity, label semantics and partition rules need to accompany a benchmark score. The source study’s DenseNet201 result is contextual evidence from its own training and split procedure, not a paired comparator sharing the present image assignments. A further development in 2026 is synthetic supervision aimed at annotation scarcity and domain shift. Asadi et al. [14] introduced CrackSegFlow, a mask-conditioned flow-matching method that generates aligned crack image–mask pairs, together with the CSF-50K benchmark. Its topology-preserving conditioning and use of crack-free backgrounds target continuity and background confounders in segmentation. This addresses data generation rather than merely selecting another classifier architecture. The present benchmark does not use synthetic images or pixel masks; therefore, these findings identify a distinct route for future segmentation studies rather than an experimentally validated improvement to the nine-category results reported here.

### 2.4. Dependence, Training Budgets and Practical Comparability

The literature motivates three separate levels of generalization: new stored variants, new source photographs and new acquisition sites. A filename-family split can keep recoverable augmentation relatives together, but different filenames can still denote repeated content or crops from the same photograph. Conversely, perceptual similarity does not prove a shared acquisition source. The parent-image structure of SDNET2018 [3] and the multi-source composition of consolidated benchmarks [10,11,12] make these distinctions important. Content screening is therefore a sensitivity check on identified relationships, not a substitute for verified parent-image or site metadata. Uncertainty estimates must also reflect the dependence unit actually used, rather than treating every related crop as an independent replication. Architectural comparisons additionally depend on training and execution conditions. Pretrained transfer learning, as considered in Omni-Crack30k [12], differs from learning all parameters from a small randomly initialized sample. At a fixed epoch count, different minibatches produce different numbers of optimizer updates, and a model still improving at the final epoch has not demonstrated its performance ceiling. Parameter count and measured latency are likewise distinct quantities: implementation, input size and host hardware affect execution. For these reasons, the present study reports its exact training budget and host-specific resource measurements, and treats family-level summaries as descriptions of selected implementations rather than universal properties of CNNs or attention models.

### 2.5. Synthesis and Position of the Present Study

The studies reviewed here show advances in multiscale features, contextual attention, boundary supervision, efficient sequence processing and dataset design. They also expose a recurring comparability problem: different targets, source relationships and training recipes can produce scores that answer different questions. The specific gap addressed here is a documented surface-category comparison that combines explicit label semantics, recoverable image-dependence checks, validation-based checkpoint selection, conditional uncertainty and training-budget sensitivity within one identified release. This is an evaluation contribution, not a claim of architectural novelty or exhaustive coverage of the field. Its interpretation remains limited to the supplied categories until independent source/site evaluation and the annotations needed for defect measurement are available.

## 3. Materials and Methods

### 3.1. Overview of Methodology

**Figure 1:**
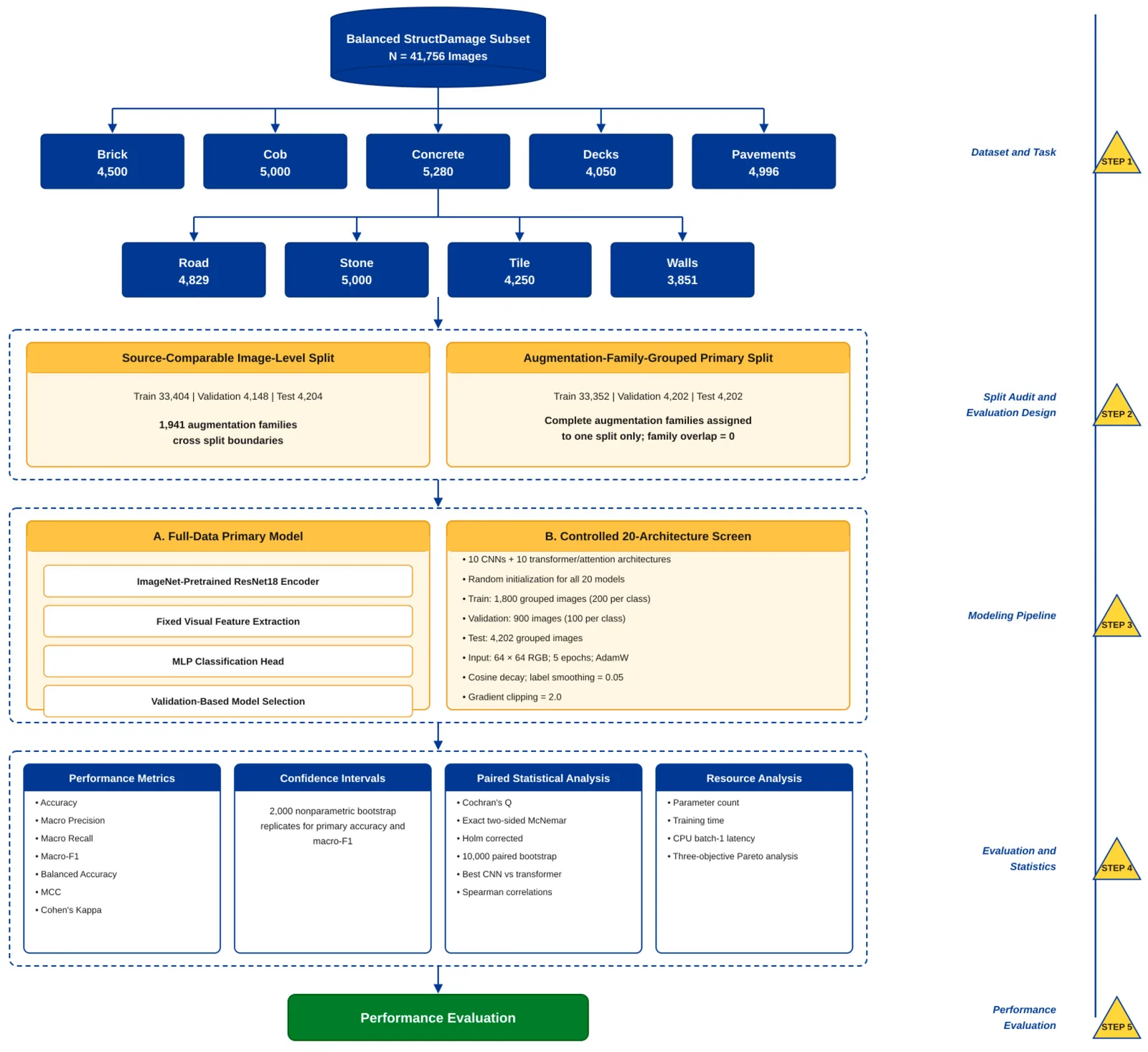
Original two-tier experimental workflow, including the initial image-level statistical diagnostics. Filename-family isolation does not guarantee zero content or source overlap. The primary component-resampling analysis, content-based screening and training-budget sensitivity are specified in Section 3.3 and Section 3.5 and evaluated in Section 4.7 and Section 4.8.

The problem is single-label classification of structural-surface imagery into nine supplied categories. The procedure begins with identifying the local release, verifying readable images and defining the evaluation unit. It then constructs an image-level sensitivity split and an augmentation-family-grouped split, trains a full-data-pretrained feature classifier, and separately evaluates twenty randomly initialized architectures under a restricted budget. Validation determines each retained checkpoint; test predictions support the reported metrics and error analysis. Figure 1 presents the original two-tier experimental workflow. Content-based dependence screening, component resampling, training-history audits and training-budget sensitivity assess the robustness and limits of the recorded results. These post hoc analyses use the existing collection and do not constitute prospective independent-site validation.

### 3.2. Dataset Acquisition and Class Composition

A classification dataset must define both the observation and the target label. Here, each readable RGB image is one observation, and its supplied folder is the single target. The experiments use the local Balanced/Balanced release of StructDamage [10]: 41,756 images across the nine categories in Table 2. This is a particular balanced derivative, not the complete public StructDamage release. The separate 49,124-image original local release is not used in these experiments. Bounding boxes, crack masks, physical scale references, severity grades and structural safety targets are not available for this nine-class task.

**Figure 2:**
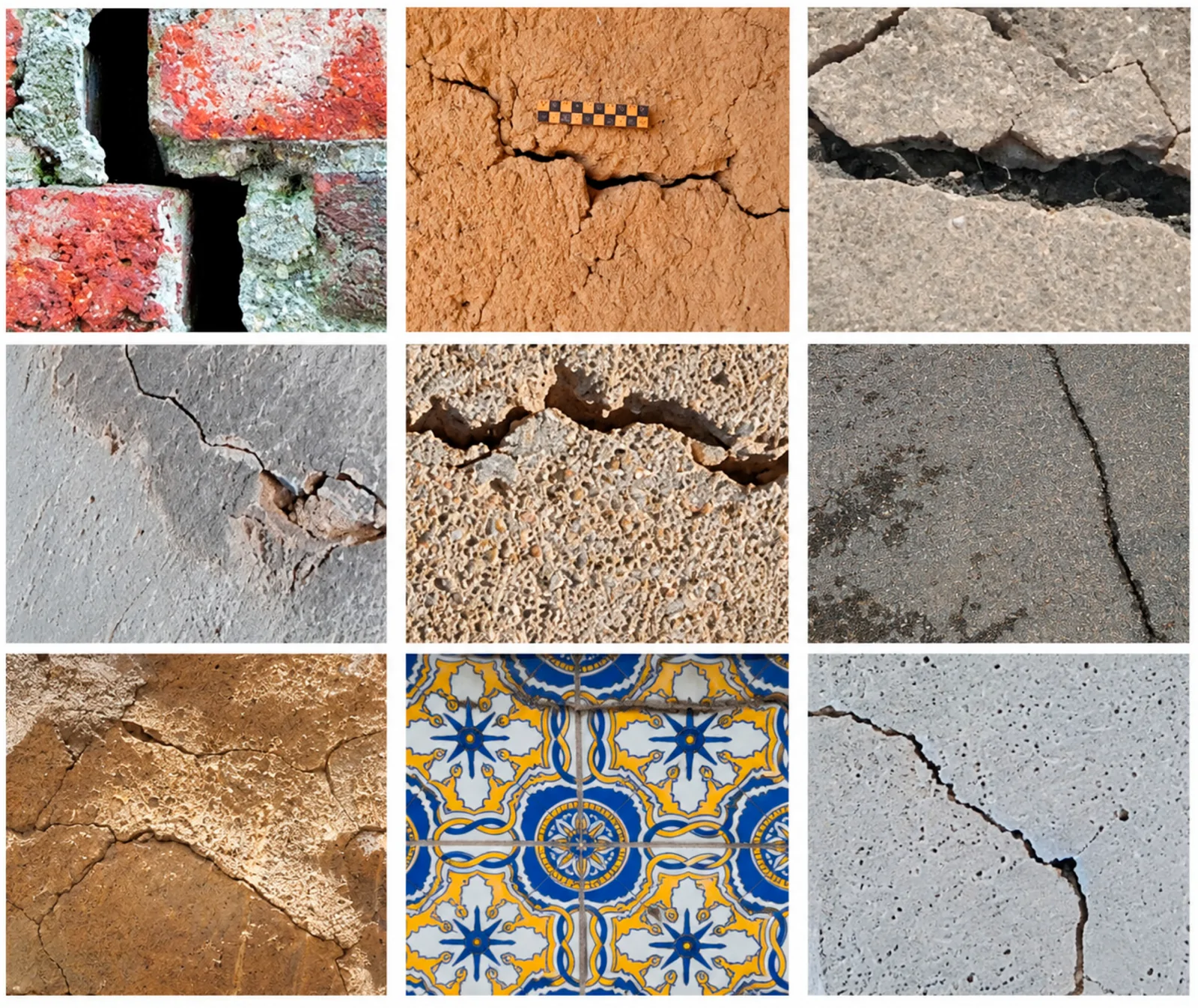
StructDamage examples: bricks, cob, concrete, decks, pavements, road, stone, tile, and walls.

Figure 2 shows actual examples of the nine supplied categories. The examples are from the grouped test partition and are illustrative, not a selection used in optimization or checkpoint choice. Category names mix material descriptions, such as brick and concrete, with surface or element context, such as decks and walls. They should therefore be interpreted as dataset categories rather than a universally exclusive material taxonomy. Surface texture, cracks, coatings and acquisition background may all be correlated with these labels.

### 3.3. Training, Validation, and Testing Datasets

Training data determine model parameters; validation data determine checkpoint selection and other stated choices; test data estimate performance after those choices. Related variants must remain together when the desired evaluation concerns new image families. The source-count-matched image-level split uses 33,404 training, 4148 validation and 4204 test images with seed 20260822. It matches the source study’s reported counts, not its unknown exact sample assignments. The filename audit identifies 1941 inferred augmentation families crossing those partitions.

For the grouped split, each lower-case filename stem is normalized by removing a terminal _aug followed by digits, optionally followed by _extra and digits, and then a terminal _extra followed by digits. The class name prefixes the normalized stem. Complete inferred families are shuffled within class with seed 20260923 and allocated as indivisible units, targeting approximately 10% of each class for validation and test sets; the remainder is the training set. Whole-family allocation can overshoot a target, so the procedure is a deterministic greedy allocation, not a global optimization. It produces 33,352/4202/4202 images and zero shared inferred filename families. Table 3 separates the protocols and their roles.

Grouping is appropriate when stored augmentation variants share a recoverable source stem, repeated crops share a known parent identifier, or sequential frames share an acquisition unit. Its advantage over a random image split is that those known relatives cannot appear in both training and testing. Its limitation is exactly the information encoded by the identifier: changing a filename does not make a photograph independent, and two crops from one structure can have unrelated stems. The resulting class counts and group assignments are supplied, so that the 33,352/4202/4202 split can be checked directly.

The content-based audit preserves the original split assignments and determines exclusions solely from image relationships, not prediction correctness. Every file receives a byte SHA-256 hash, a SHA-256 hash of decoded RGB pixels and image dimensions, and a 64-bit horizontal difference hash. The latter uses an RGB image resized to 9 × 8 and converted to grayscale, with one bit for each adjacent horizontal comparison. Connected components join any equal inferred filename family, equal byte hash, equal decoded-pixel hash, or difference hashes at a Hamming distance of at most 3, including matches across classes. Four disjoint 16-bit blocks generate candidates; full Hamming distance confirms each candidate. A test image is retained for the content-screened sensitivity analysis only if its complete component contains no original training or validation image. Exact matching detects repeated content; perceptual matching is a conservative similarity screen with possible false matches and misses. It does not recover complete parent-image or site provenance.

ImageNet is a labeled natural-image dataset used for pretraining, not a classification algorithm [15]. The full-data pipeline uses the locally cached official ResNet18 ImageNet weights [16] as a frozen encoder. The actual implementation resizes the shorter image side to 176 pixels with bilinear interpolation, takes a 160 × 160 center crop, and applies channel means (0.485, 0.456, 0.406) and standard deviations (0.229, 0.224, 0.225). This is the recorded study preprocessing, not the unmodified default weight transform. The 512-dimensional features are cached in float16 and converted to float32 for training. The classifier is LayerNorm(512), Linear(512,384), GELU, dropout 0.15, and Linear(384,9). AdamW [17] uses a learning rate of 0.002, weight decay of 0.0002, batch size of 512 and cross-entropy label smoothing of 0.03. The cosine schedule is configured for 100 epochs with minimum rate of 0.00002. Training stops after 15 non-improving validation macro-F1 epochs once at least 25 epochs have run; an improvement must exceed 0.00001. The grouped head runs 39 epochs and selects epoch 24. Only the head is optimized; the encoder and its batch-normalization statistics remain fixed.

For the separate architecture screen, only two pretrained weight sets were locally cached, so an all-pretrained comparison could not be supported offline. All twenty screen models instead use random initialization and nine-class outputs. The original sample contains 200 training images and 100 validation images per class (1800 and 900 total), with all 4202 grouped test images. The common cache first selects 600 training and 200 validation images per class with NumPy seed 20261024; the original screen uses the first 200 and 100 cached members per class. 

Screen images are converted to RGB and resized to 64 × 64 with bilinear interpolation. Online training augmentation uses independent horizontal flips with a probability of 0.5 and vertical flips with a probability of 0.2, brightness and contrast factors uniformly sampled from 0.9 to 1.1, clipping to [0, 1], and the same ImageNet channel normalization. Validation and test images receive no random augmentation. AdamW uses a learning rate of 0.0008 and a weight decay of 0.0005; cross-entropy label smoothing is 0.05, gradient-norm clipping is 2.0, and cosine decay reaches 0.00002 over five epochs. Batch size is 12 for DenseNet121, ResNet34, Swin-Tiny, MaxViT-Tiny, DeiT-III-Small/16 and CaiT-XXS24, and 32 for the others. Incomplete training batches are dropped: the original runs therefore perform 150 or 56 optimizer updates per epoch, respectively. Equal epochs and data do not imply equal numbers of updates or equal computational budgets. The highest validation macro-F1 checkpoint is retained, with the earlier checkpoint retained on an exact tie. Seed 20260822 is used for model initialization and training.

The training-data sensitivity analysis retains the same original validation and test sets, draws no new test data, and increases the nested training subset to 600 images per class (5400 total). Every architecture is restarted from random initialization under the same five-epoch optimizer and augmentation settings, with the original per-architecture minibatch policy. Thus, the change combines more distinct training images with more optimizer updates at fixed epoch count; it is not a fixed-update causal data-size experiment. The full-data-pretrained pipeline already uses all 33,352 grouped training images. The twenty-model screen and its enlarged subset are not described as full-data end-to-end training. Recorded histories assess whether the final epoch still improves validation performance; no convergence claim is inferred merely from completion of a schedule.

### 3.4. Evaluated Architectures

The twenty architectures were chosen to cover established residual and dense CNNs, mobile-efficient convolution, modern convolutional design, patch-token attention, hierarchical attention and mobile hybridization. They are not a random sample of all possible networks. Each architecture retains its native topology with a nine-class output; Table 4 identifies its implementation, parameter count and minibatch.

The CNN mechanisms are summarized by the residual networks [16], ConvNeXt [18], DenseNet [19], EfficientNet [20], GhostNet [21], MNASNet [22], MobileNetV2 [23], MobileNetV3 [24] and RegNet [25]. The transformer/attention group covers CaiT [26], DeiT III [27], LeViT [28], MaxViT [29], MobileViT [30], PVT v2 [31], PiT [32], Swin [33], ViT [34] and XCiT [35]. Table 5 explains the operating principle of every named model. Architecture names denote the instantiated implementation variants, not reproductions of each source paper’s training recipe.

**Figure 3:**
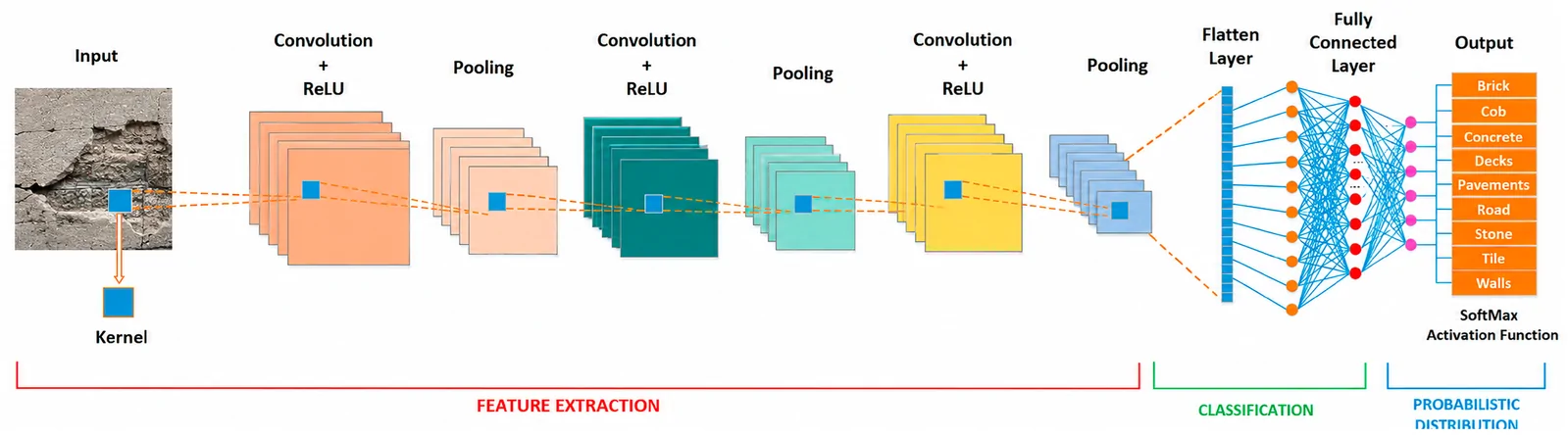
Conceptual CNN schematic for nine-class image classification. The evaluated implementations retain their own pooling, block and classifier topology.

Figure 3 illustrates convolutional feature extraction followed by image classification. It is a conceptual schematic, not the exact topology of all ten CNNs: some models use global pooling rather than the illustrated flattening step. Likewise, attention architectures differ in their tokenization and downsampling. At 64 × 64 resolution, a 16 × 16 patch model begins with only 16 spatial tokens; this setting is a substantive experimental constraint, not a neutral replacement for the resolutions used in the architecture papers.

Table 6 summarizes the distinct purposes, data allocations and input settings of the two experimental tiers.

### 3.5. Performance Evaluation and Statistical Analysis

A confusion matrix counts how supplied classes are mapped to predicted classes. Let C_ij_ be the number of images with true class i and predicted class j, N the number of evaluated images, and K = 9. Its diagonal contains correct predictions; row-normalized values describe class recall and the distribution of errors from each true class. In a one-versus-rest view for class k, TP_k_ = C_kk_, FN_k_ is the off-diagonal row sum, and FP_k_ is the off-diagonal column sum. Accuracy weights every image equally; macro-averaged scores weight the nine classes equally [36]. Equations (1)–(6) define the metrics, with zero-denominator precision or F1 assigned zero. Balanced accuracy equals macro recall for this multiclass task. Multiclass Matthews correlation coefficient (MCC) summarizes agreement using the complete matrix [37]; Cohen’s kappa adjusts observed agreement for the agreement implied by the two class marginals [38]. In Equations (5) and (6), c is the sum of diagonal counts, t_k_ and p_k_ are the true and predicted class totals, p_o_ = c/N, and p_e_ = Σ_k_t_k_p_k_/N^2^.

(1) $$\mathrm{Accuracy}=\frac{\sum_{k}C_{\mathrm{kk}}}{N}$$

(2) $$P_{k}=\frac{{\mathrm{TP}}_{k}}{{\mathrm{TP}}_{k}+{\mathrm{FP}}_{k}}\;\;R_{k}=\frac{{\mathrm{TP}}_{k}}{{\mathrm{TP}}_{k}+{\mathrm{FN}}_{k}}$$

(3) $${F1}_{k}=\frac{2{\mathrm{TP}}_{k}}{2{\mathrm{TP}}_{k}+{\mathrm{FP}}_{k}+{\mathrm{FN}}_{k}}\;\;\mathrm{Macro}-F1=\frac{\sum_{k}{F1}_{k}}{K}$$

(4) $$\mathrm{Macro precision}=\frac{\sum_{k}P_{k}}{K}\;\;\mathrm{BA}=\mathrm{Macro recall}=\frac{\sum_{k}R_{k}}{K}$$

(5) $$\mathrm{MCC}=\frac{\mathrm{cN}-\sum_{k}p_{k}t_{k}}{\sqrt{\left(N^{2}-\sum_{k}p_{k}^{2}\right)\left(N^{2}-\sum_{k}t_{k}^{2}\right)}}$$

(6) $$\kappa =\frac{p_{o}-p_{e}}{1-p_{e}}$$

For the actual DenseNet121 wall predictions in Section 4.6, the one-versus-rest matrix is [[224, 161], [22, 3795]], with rows [wall, not wall] and columns [predicted wall, predicted not wall]. Hence, wall precision is 224/(224 + 22) = 0.9106, recall is 224/(224 + 161) = 0.5818, and F1 is 448/(448 + 22 + 161) = 0.7100. The 159 wall-to-deck errors are part of the 161 false negatives; the remaining two are predicted concrete and stone. This worked matrix uses observed counts, not a fabricated illustrative experiment.

Image-level bootstrapping [39] is reported as a secondary sensitivity analysis. Because augmented and related images are dependent, the primary uncertainty analysis resamples complete content-audit components [40]. For the 4202-image grouped test set, 1703 components are sampled with replacement 5000 times using seed 20260914. All images within each sampled component are retained; 2.5th and 97.5th percentiles define conditional intervals. Identical component draws are used across models for paired accuracy differences. The screened 4102-image set contains 1661 components and uses 2000 resamples. Components are operational hash/filename units, not verified sites; these intervals do not include model-training-seed uncertainty or uncertainty about source provenance.

Cochran’s Q [41] and exact two-sided McNemar tests [42], with Holm correction across nineteen champion-versus-alternative contrasts [43], reproduce the original image-level diagnostics. They are treated as secondary because their usual independent-unit interpretation is not satisfied by related images. Selection of a champion from observed test scores also makes subsequent champion contrasts exploratory. Spearman correlation [44] describes accuracy versus resource measures and ranking agreement between data budgets. Tests and intervals describe the recorded runs; they are not independent replications of architecture families.

### 3.6. Reproducibility and Computational Environment

Supplementary S1 documents the relative-path split manifests, saved and recomputed test predictions, training histories, model settings, analysis and training code, software-version records, hash audits and output checksums. The recorded environment for the audit and sensitivity analyses is Python 3.11.9, PyTorch 2.5.1+cpu, torchvision 0.20.1+cpu, timm 1.0.28, NumPy 2.4.6, pandas 3.0.5, SciPy 1.17.1, scikit-learn 1.9.0, Pillow 12.3.0 and Matplotlib 3.11.1 on Windows. These environment records are distinguished from the archived original outputs. Computation was performed on a Dell computer (Dell Technologies, Round Rock, TX, USA) equipped with an Intel Core i5-1235U CPU with 12 logical processors; no GPU or newly downloaded pretrained models were used. The original screen requested 10 Torch threads; the enlarged-data runs request four to limit local resource contention. The original latency measurements are reported as host-specific diagnostics and are not pooled with timings from the enlarged-data runs.

The primary encoder weight file is resnet18-f37072fd.pth, with SHA-256 f37072fd47e89c5e827621c5baffa7500819f7896bbacec160b1a16c560e07ec. Supplementary S1 records original checkpoint and input-manifest digests, distinct run directories and validation-selected epochs. Dataset-relative paths identify the inputs independently of local directory names. The original runs, post hoc screening and enlarged-data runs are identified separately to preserve their experimental provenance.

## 4. Results

The experiments evaluate how reliably the supplied surface labels can be predicted under the two specified regimes. Section 4.1, Section 4.2, Section 4.3, Section 4.4, Section 4.5 and Section 4.6 present the original full-data feature classifier and the short architecture screen using their saved predictions. Section 4.7 and Section 4.8 report the content-based audits and sensitivity computations. Pretraining, input resolution and training-set size differ between tiers, so a 98.88% feature-pipeline result is not compared as though it arose from the same experiment as the 88.34% screening result. Test-based rankings are descriptive; model checkpoints were selected using validation data.

### 4.1. Full-Data Classification Under the Two Original Protocols

Table 7 reports the full-data results. The source-count-matched image-level pipeline obtains 99.2388% accuracy and 0.99160 macro-F1 on 4204 images. The filename-family-grouped pipeline obtains 98.8815% accuracy, 0.98813 macro-F1, 0.98818 balanced accuracy, 0.98741 MCC and 0.98740 kappa on 4202 images. Its primary 95% component-bootstrap accuracy interval is 98.42–99.26%. The original image-bootstrap interval of 98.55–99.19% is retained in Supplementary S1 for provenance, not as the primary uncertainty estimate. The 0.3573-percentage-point difference between the two protocols is not a causal estimate of leakage inflation: their test membership also differs. The 98.6203% DenseNet201 source result [10] is contextual only, not a directly paired baseline. Residual content overlap and its screened sensitivity result are reported in Section 4.7.

**Figure 4:**
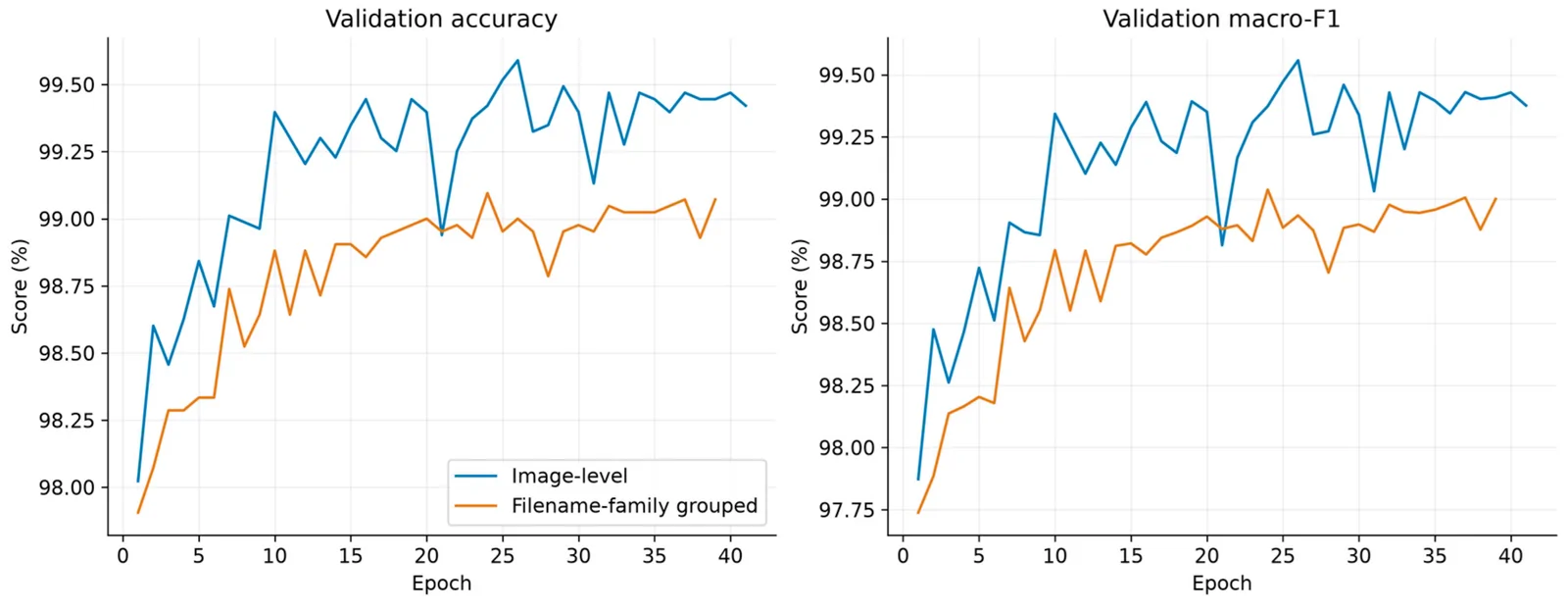
Recorded validation accuracy and macro-F1 for the two full-data classifier-head runs; these are not the twenty-architecture training curves.

Figure 4 shows the actual validation histories of the two classifier heads. These curves describe optimization over fixed pretrained features, not convergence of twenty end-to-end architectures. The grouped head selects epoch 24 and stops at epoch 39 under the recorded patience rule. Its high later validation scores support the stability of this particular head-training run; they do not establish independent-site generalization or the sufficiency of five epochs for the architecture screen.

**Figure 5:**
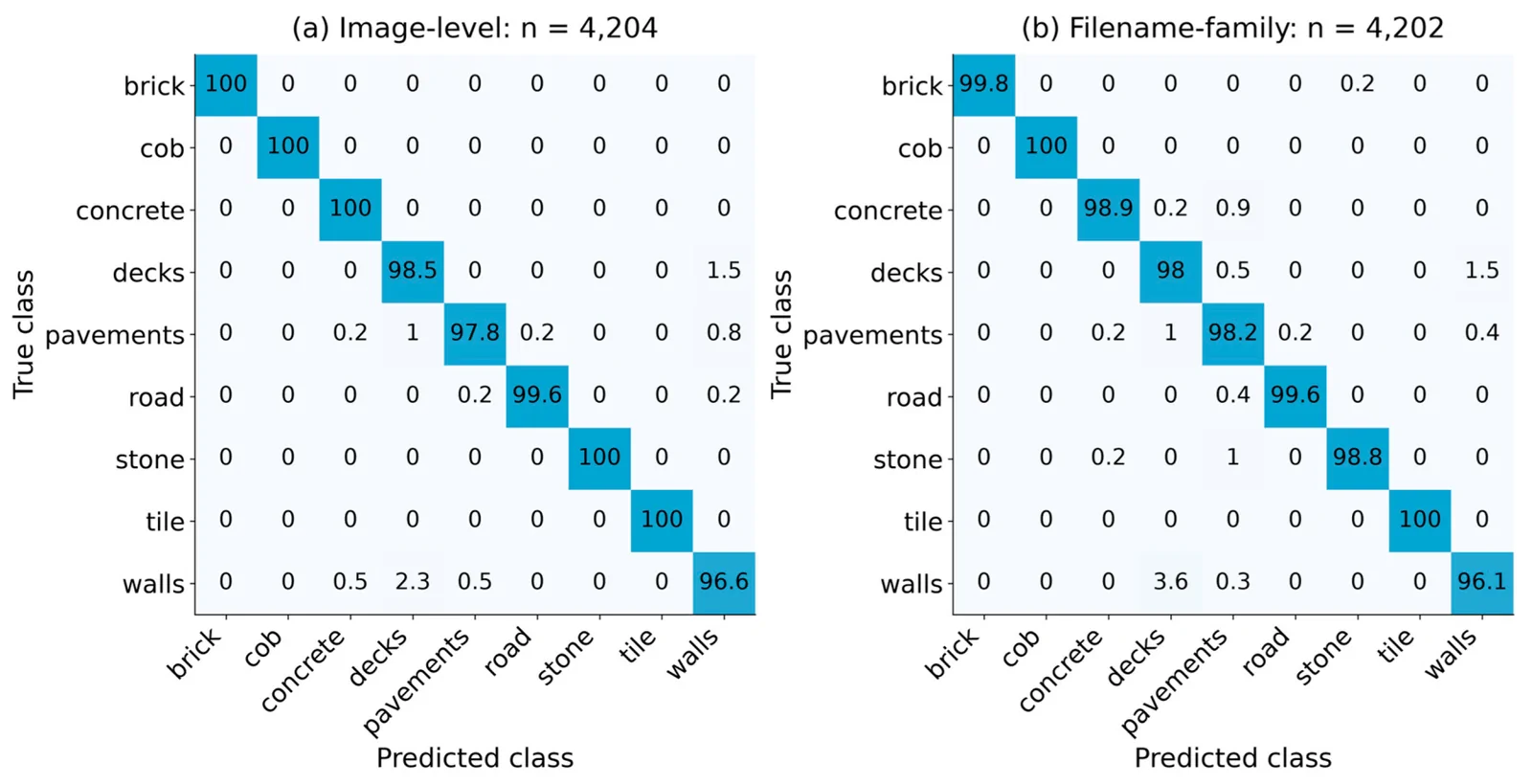
Full-data confusion matrices. Values are within-true-class percentages; each panel uses its own stated test partition.

Figure 5 shows the full-data confusion matrices for the two partition protocols. Under filename-family grouping, the residual errors are concentrated in a subset of surface classes rather than uniformly spread across all nine labels. The matrix is strongly diagonal, but this observation must be read with the dependence audit in Section 4.7. A diagonal matrix alone does not establish that the test images represent new sites or independent source photographs.

### 4.2. Class-Wise Performance of the Grouped Primary Model

Table 8 gives precision, recall, F1 and support for the grouped full-data model. Per-class F1 ranges from 0.9660 for decks to 1.0000 for cob and tile; walls have an F1 0.9699 and the lowest recall, 0.9610. The macro-F1 of 0.9881 is close to the weighted F1 of 0.9888, but this does not eliminate related-image dependence. These class-specific measurements characterize the supplied labels and should not be interpreted as probabilities of detecting dangerous damage.

**Figure 6:**
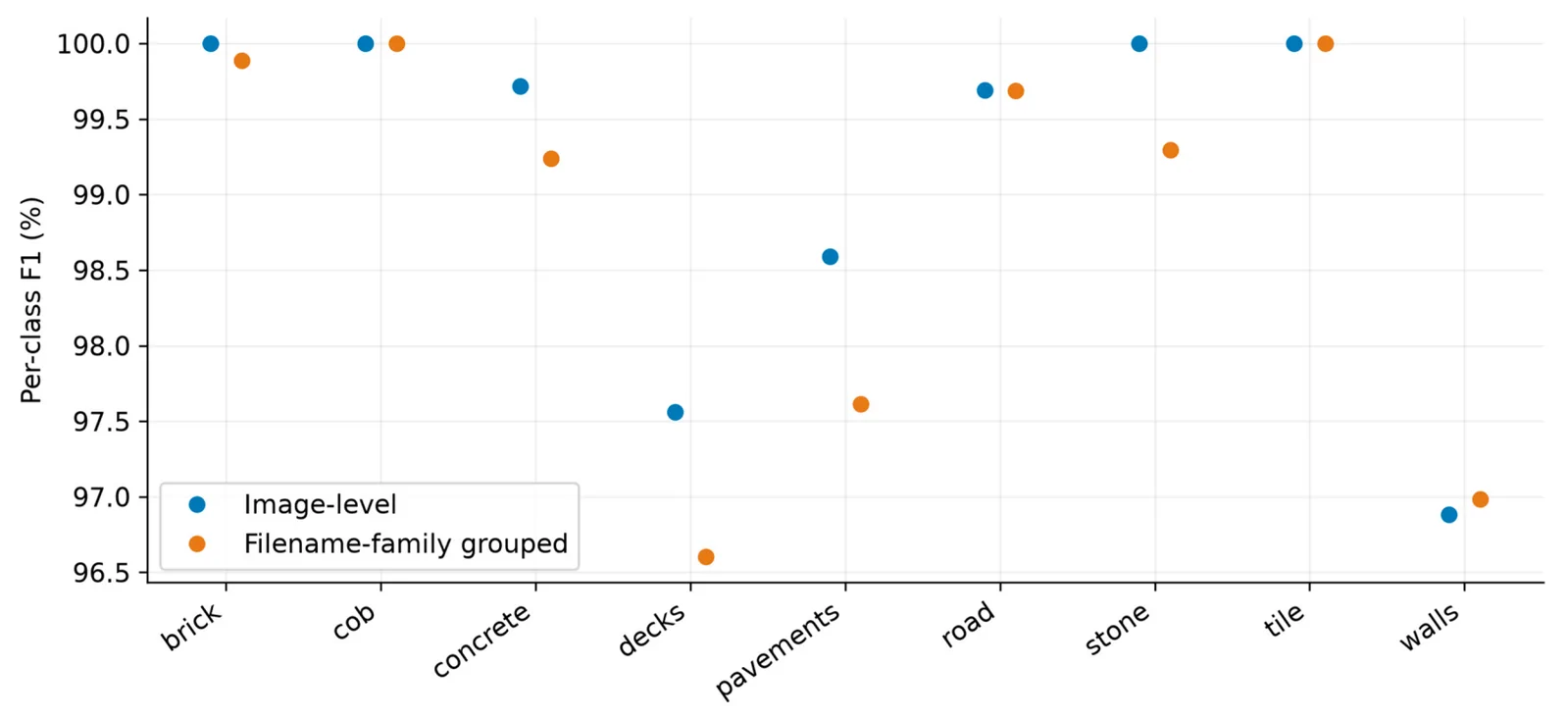
Per-class F1 of the two original full-data protocols, which have different test memberships.

Figure 6 compares the per-class F1 profiles of the original two protocols. Their patterns are similar, with decks, pavements and walls remaining important residual-error categories. The two profiles use different test membership and therefore describe protocol sensitivity, not paired per-image improvements.

**Figure 7:**
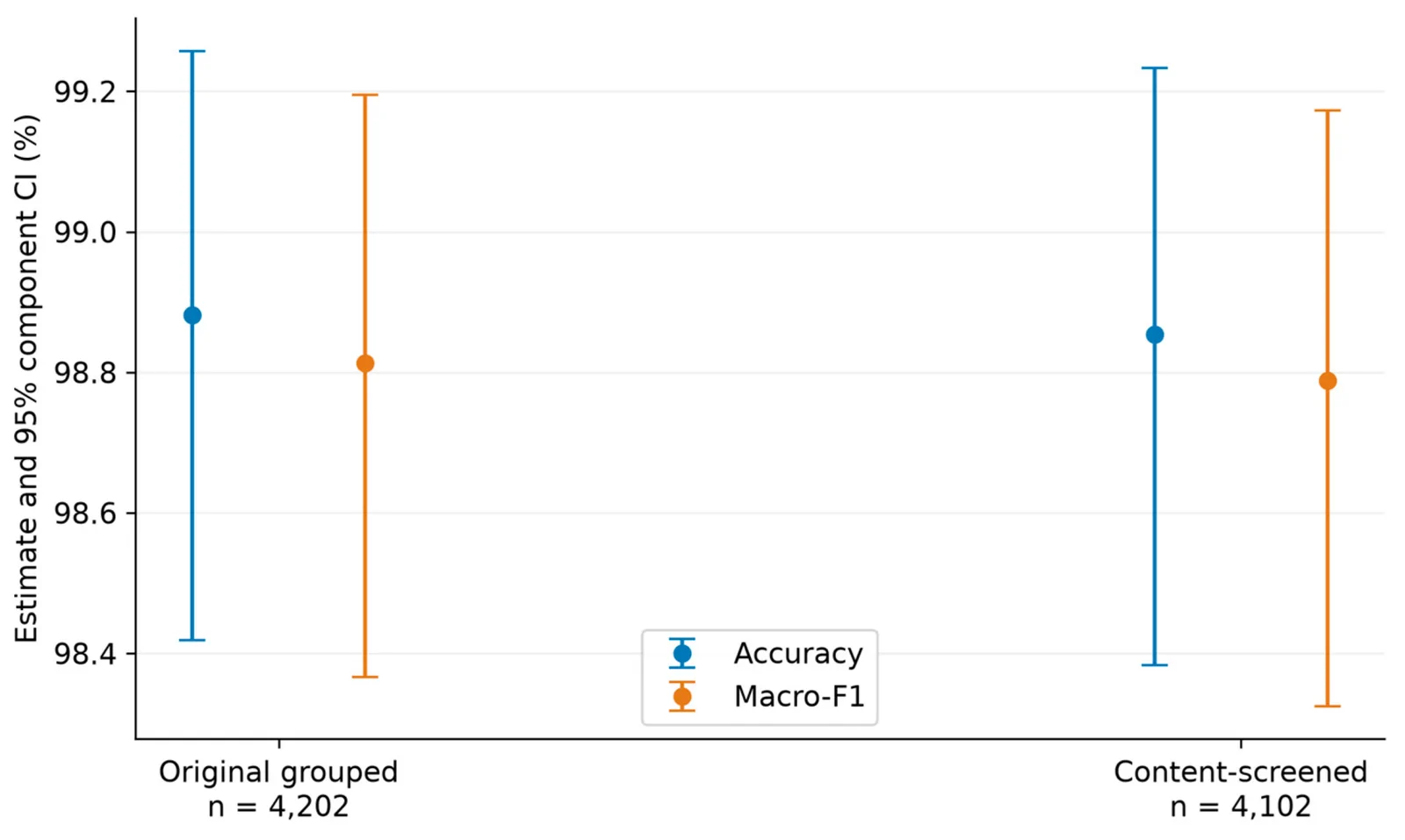
Grouped pretrained ResNet18 estimates before and after content screening, with percentile component-bootstrap intervals. This is a post hoc sensitivity analysis, not an external validation.

Figure 7 contrasts the grouped full-data result before and after content screening using component-resampling intervals. The screened accuracy is 98.85% on 4102 images, close to the 98.88% original grouped point estimate. This small numerical change supports stability under the implemented screening rule, but does not prove absence of source-level dependence. Confidence-interval overlap is not used as a significance test.

### 4.3. Comparative Performance of the Twenty Architectures

Table 9 reports all twenty original screening results. Accuracy ranges from 24.13% to 88.34% and macro-F1 from 0.1642 to 0.8765. DenseNet121 ranks first with 88.34% accuracy, followed by ResNet34 with 87.24% and ResNet18 with 86.72%. XCiT-Tiny is the best-performing transformer/attention model in this run with 84.75%, fourth overall. These are descriptive test rankings under random initialization, 1800 training images, 900 validation images, 64 × 64 inputs and five epochs, with unequal minibatches. They are not estimates of fully trained performance ceilings. The particularly low Swin-Tiny result is a configuration-specific observation, not evidence that shifted-window attention is generally unsuitable for structural imagery.

**Figure 8:**
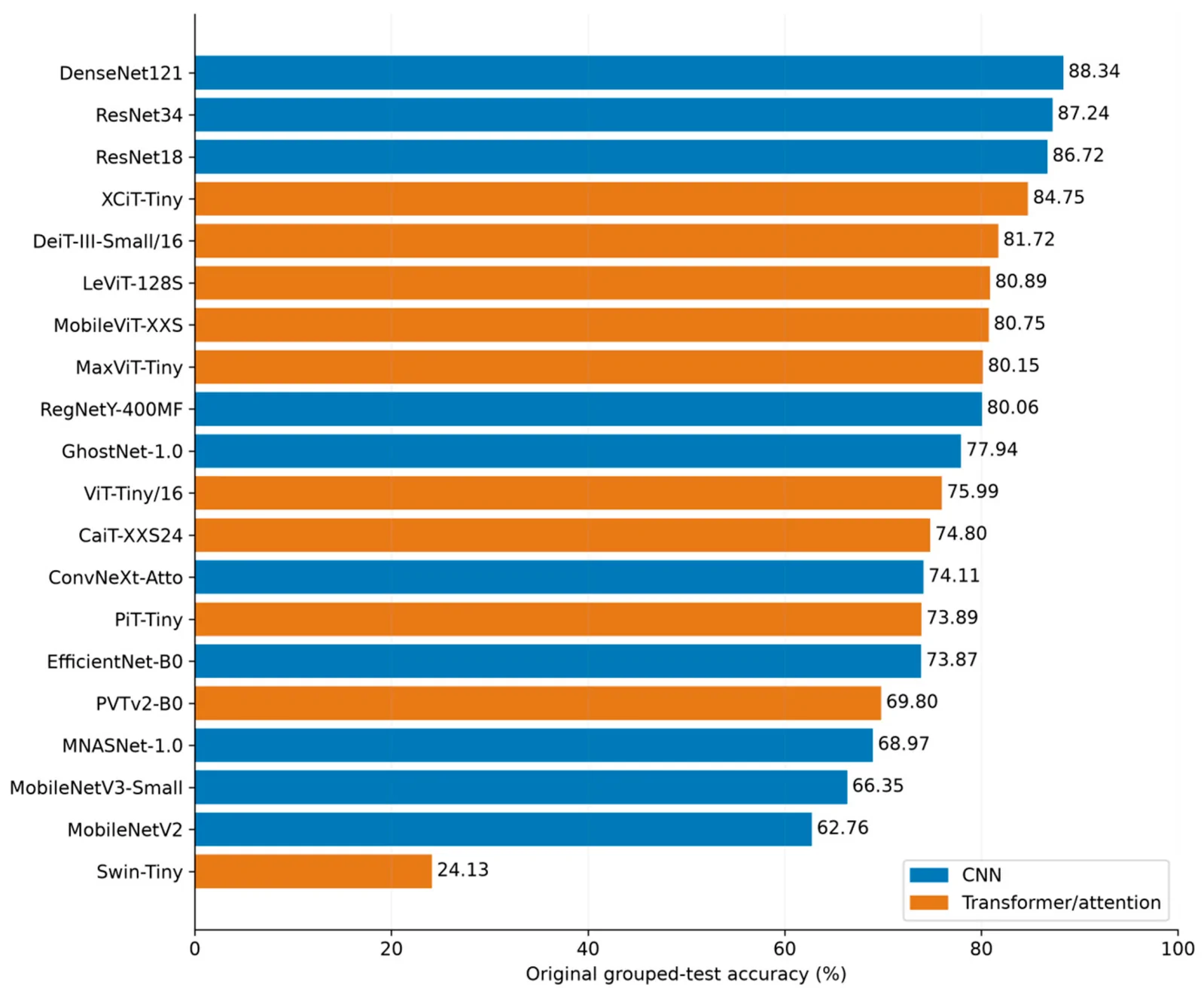
Original 20-model grouped-test accuracy ranking; colors identify CNN and transformer/attention implementations, not universally superior families.

Figure 8 displays the original accuracy ordering. Although CNNs occupy the first three positions, both groups occur throughout the ranking. Family membership alone does not determine performance. The screening order is assessed under an increased training-data budget in Section 4.8 rather than assumed to be stable.

### 4.4. CNN Versus Transformer/Attention Families and Paired Statistical Comparison

Table 10 summarizes the ten deliberately selected models in each family. Mean accuracy is 76.63% for CNNs and 72.69% for transformer/attention models, whereas the respective medians are 76.02% and 78.07%. The low Swin-Tiny score strongly affects the transformer/attention mean. These are descriptive summaries of the selected models, not a statistical sample of all architectures. Mean parameter counts are 6.15 M and 11.92 M, and original median batch-1 CPU latencies are 34.90 ms and 67.31 ms, respectively.

Table 11 gives the DenseNet121–XCiT-Tiny contrast on the common original grouped test set. The accuracy difference is 3.59 percentage points; the paired component-bootstrap 95% interval is 2.19–5.07 percentage points. The original image-resampling interval was 2.81–4.40 percentage points, but it treated related test images as independently resampled units. The component analysis retains all images in a sampled dependence component and uses identical component draws for both models. Because the best-in-family models were identified after viewing test results, the contrast remains exploratory rather than a preregistered confirmatory family test.

As a secondary diagnostic, image-level Cochran’s Q is 13,299.10 with 19 degrees of freedom and a numerically underflowing *p*-value. Exact image-level McNemar comparisons with Holm correction are reported in Table 12. All nineteen adjusted image-level tests reject at 0.05, including DenseNet121 versus ResNet34 at approximately 0.008. However, the images include related variants, and the reference model was selected from test performance. These tests are retained as secondary descriptive diagnostics; multiplicity correction does not repair clustering or post-selection. The component-resampling contrasts in Supplementary S1 provide the dependence-aware effect intervals.

The dependence-aware interval is −0.36 to 2.44 percentage points against ResNet34 and −0.07 to 3.28 percentage points against ResNet18. Both include zero, unlike the corresponding image-level significance declarations. Thus, this single-run evidence does not resolve DenseNet121’s advantage over the two closest CNN competitors after accounting for the implemented image components.

**Figure 9:**
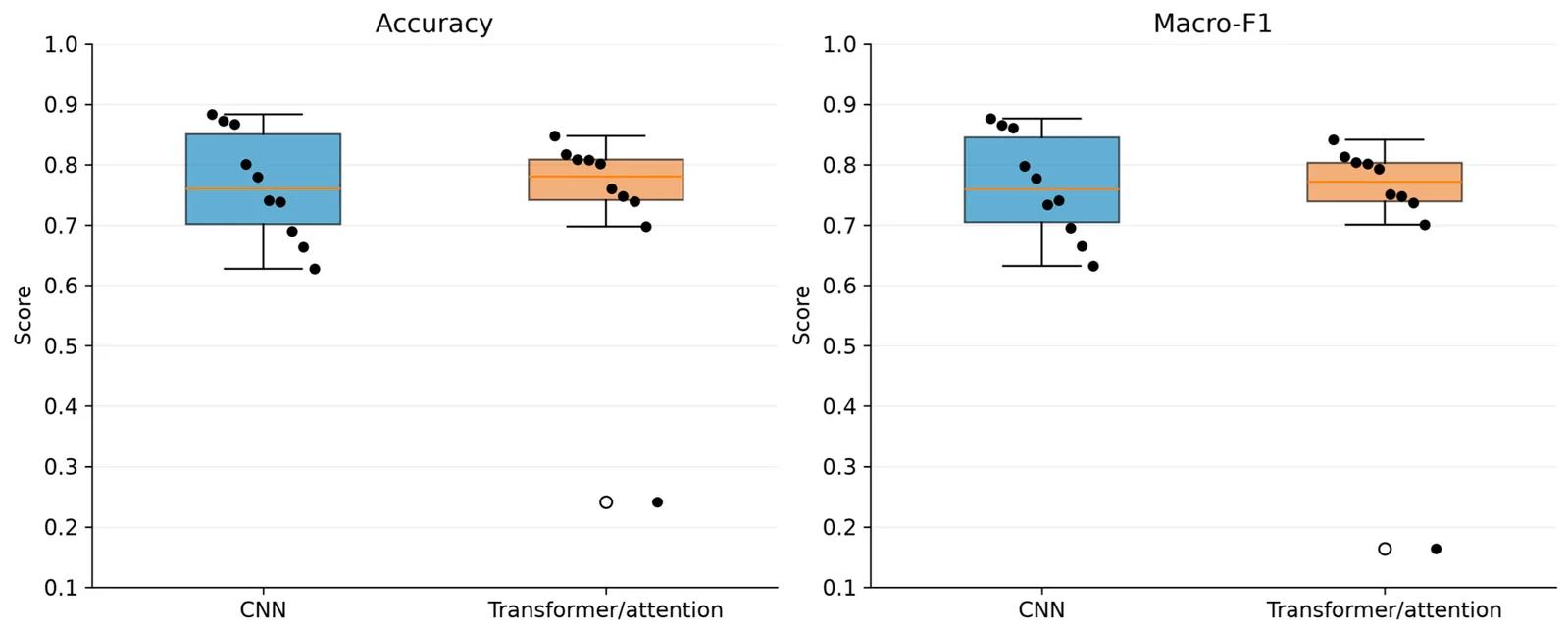
Within-group distributions for the twenty original screening runs; individual model points remain visible. Black circles represent the individual model results, while the boxplots show the corresponding group distributions.

Figure 9 displays the individual models within each family alongside their accuracy and macro-F1 distributions. The individual points are retained because each group contains only ten intentionally selected architectures. The differing means and medians primarily describe this particular set and budget; they do not support a universal CNN-versus-transformer/attention claim.

### 4.5. Accuracy, Model Complexity, and Computational Efficiency

**Figure 10:**
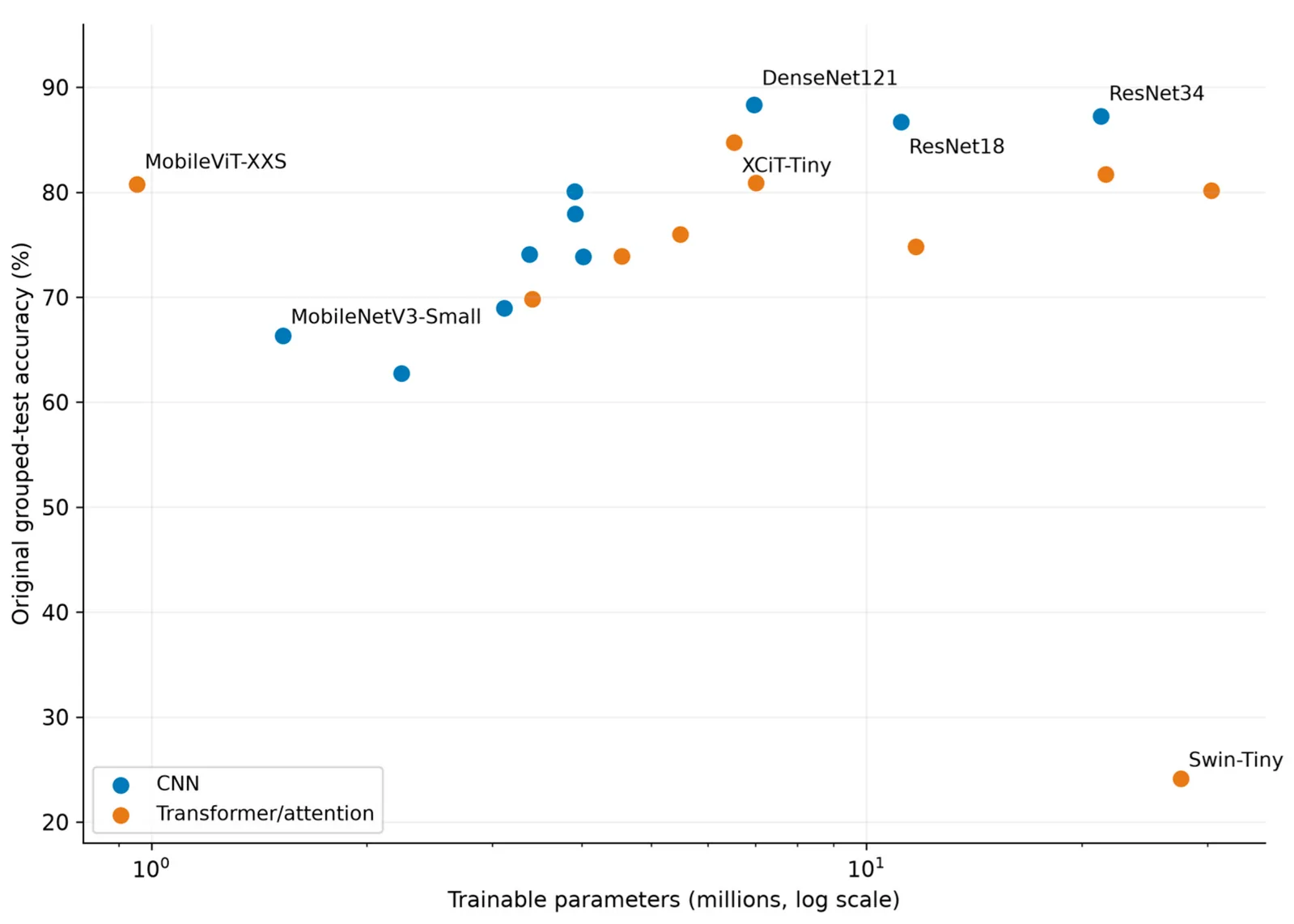
Original screening accuracy versus parameter count. The logarithmic horizontal scale accommodates the different model sizes. Each point represents one evaluated model, with colours indicating the model family. Selected representative architectures are labelled to aid interpretation; the remaining points are intentionally left unlabeled to preserve figure readability.

The original resource analysis shows no simple monotonic relation between size and accuracy: Spearman’s ρ is 0.438 for log10 parameter count versus accuracy (*p* = 0.054), and 0.162 for measured CPU latency versus accuracy (*p* = 0.494). Figure 10 shows the parameter trade-off. DenseNet121 combines 88.34% accuracy with 6.96 M parameters and 65.53 ms per image in the original latency check; ResNet18 obtains 86.72% with 15.51 ms. These timing checks contain only ten measured forward passes after three warm-ups and are host- and implementation-specific. They exclude image decoding and do not establish a guaranteed real-time deployment rate.

**Figure 11:**
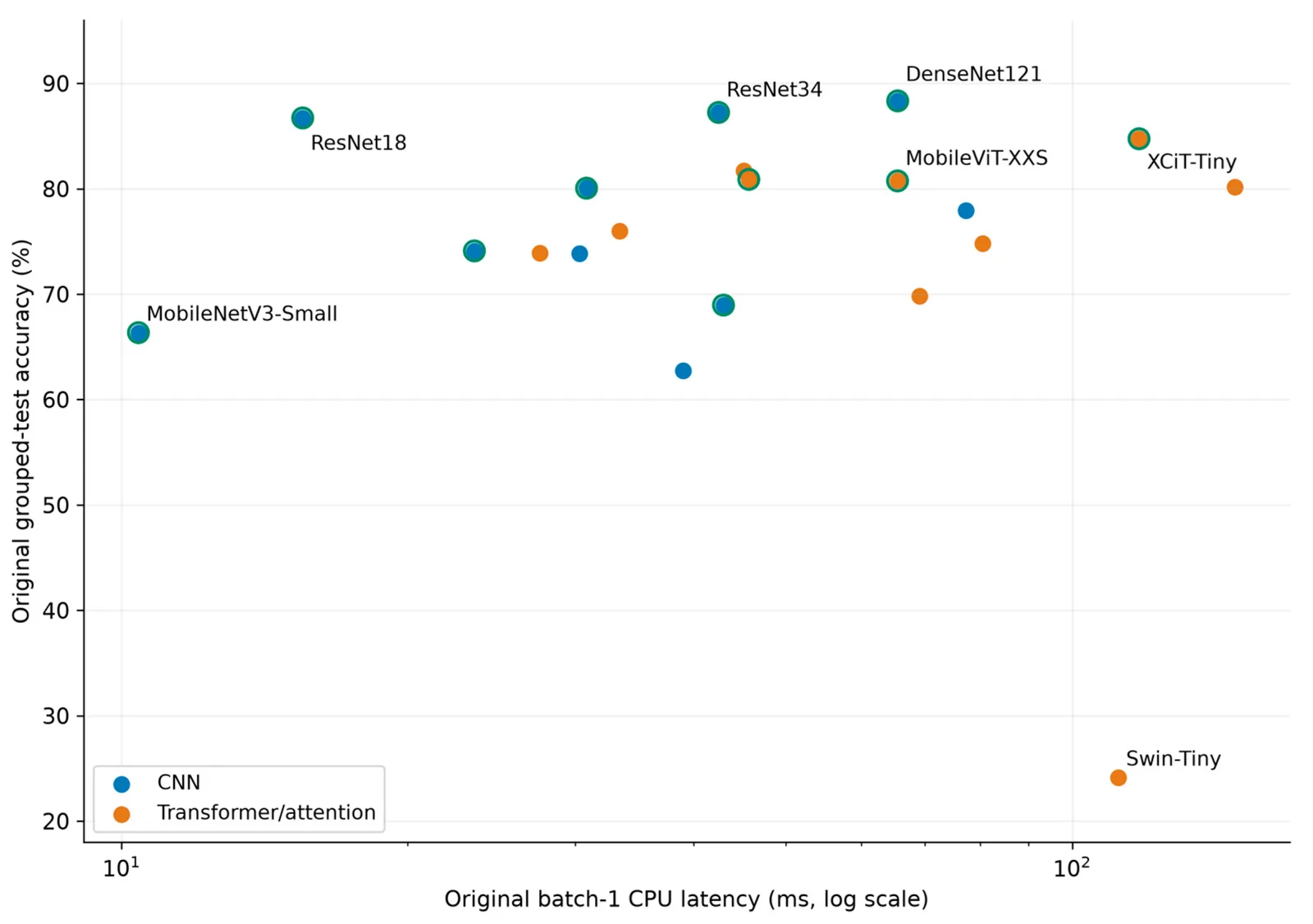
Original screening accuracy versus batch-1 CPU latency; rings identify the observed three-objective non-dominated set.

Figure 11 shows the latency trade-off. The compact MobileViT-XXS has 0.95 M parameters but a recorded latency of 65.51 ms, while MobileNetV3-Small records 10.42 ms with 66.35% accuracy. Thus, parameter storage and CPU execution cost are separate constraints. A deployment choice must also consider error type, operating resolution and target hardware, rather than selecting the smallest file or highest accuracy alone.

Ten models are non-dominated on the observed combination of higher accuracy, fewer parameters and lower latency: DenseNet121, ResNet34, ResNet18, XCiT-Tiny, LeViT-128S, MobileViT-XXS, RegNetY-400MF, ConvNeXt-Atto, MNASNet-1.0 and MobileNetV3-Small. Membership in this measured Pareto set is not a deployment endorsement: the figures contain single-run accuracy and short timing checks, with no field operating cost validation.

### 4.6. Class-Wise Error Analysis of the Screening Champion

**Figure 12:**
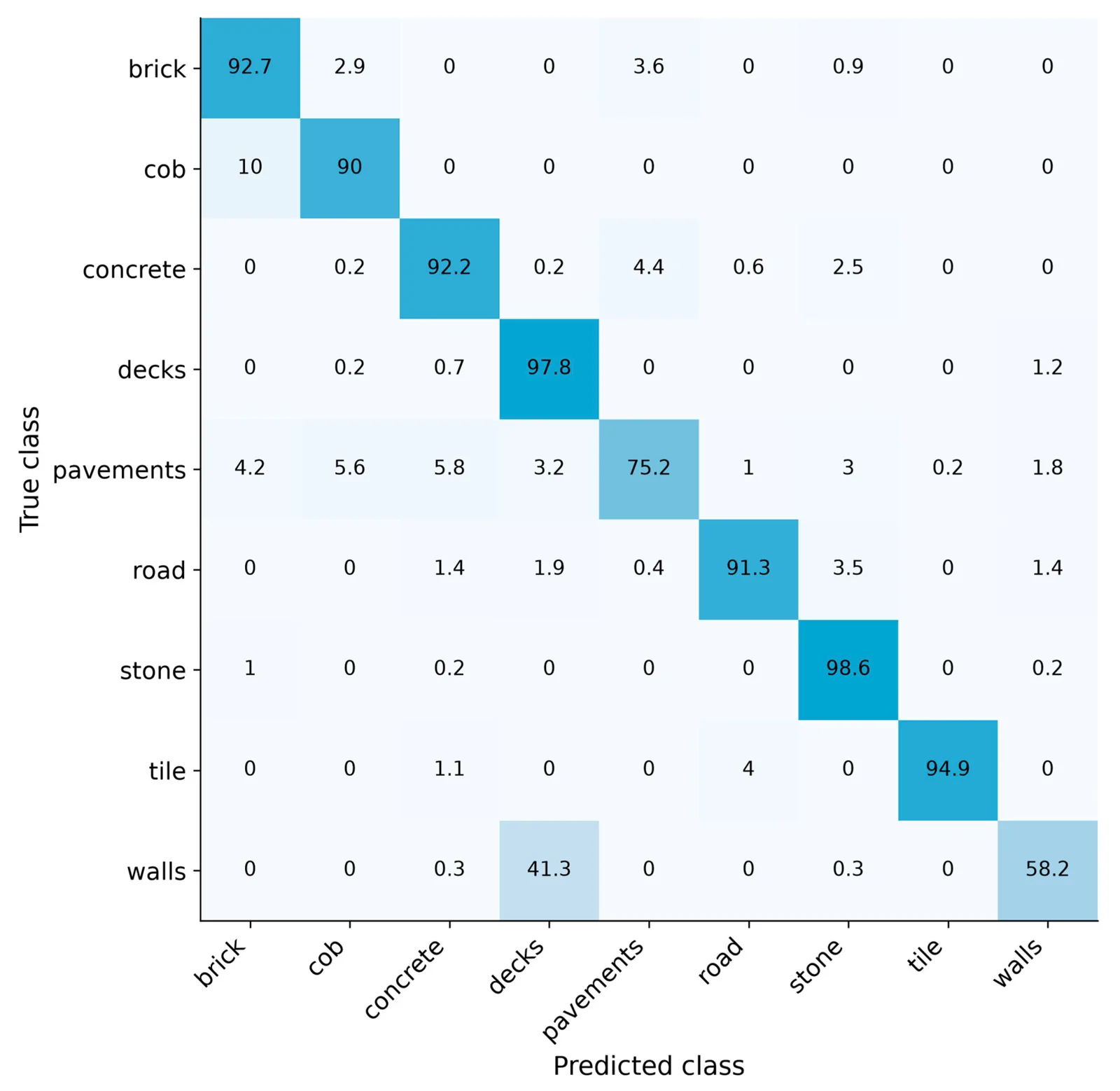
DenseNet121 original grouped-test confusion matrix. Values are row percentages; the wall-to-deck cell corresponds to 159 of 385 wall images.

Figure 12 shows DenseNet121’s original confusion matrix. The dominant asymmetry is 159 of 385 wall images predicted as decks (41.30%), compared with only 5 of 406 deck images predicted as walls (1.23%). Wall recall is 224/385 = 58.18%, while deck recall is 397/406 = 97.78%. The wall-to-deck errors constitute 159 of the model’s 490 errors (32.45%). The worked one-versus-rest matrix in Section 3.5 uses the same actual wall predictions, connecting the metric definitions to the observed error.

**Figure 13:**
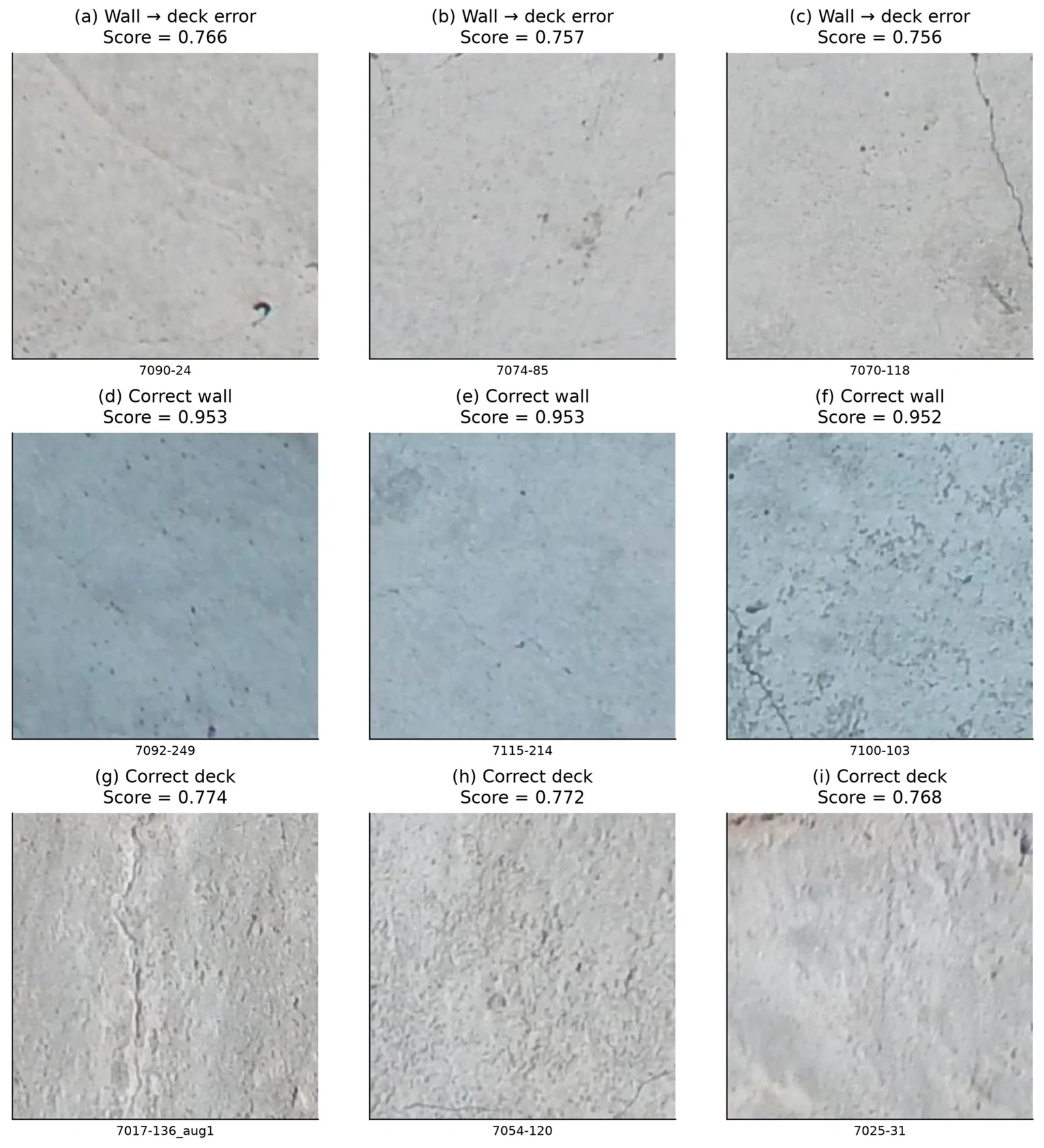
Actual, component-distinct DenseNet121 examples: (**a**–**c**) wall-to-deck errors, (**d**–**f**) correctly classified walls, and (**g**–**i**) correctly classified decks. Scores are uncalibrated maximum softmax outputs, not probabilities of structural damage. Images retain their original content.

Figure 13 places three high-confidence, component-distinct wall-to-deck errors beside three correctly classified walls and three correctly classified decks. Selection is post hoc and stratified by error type for illustration, not an estimate of typical image frequency. The image manifest records every filename and score. These cropped views often lack scene-level orientation or element context, so an image can plausibly share local concrete texture with more than one supplied context label. This visual interpretation does not establish that the annotations are wrong. No expert relabeling, segmentation-ground-truth comparison or controlled background-ablation experiment is available.

The error is systematic within the recorded short-budget predictions and asymmetric: it persists for the same 159 wall images after content screening. Comparable wall-to-deck errors occur in XCiT-Tiny (148/385) and randomly initialized ResNet18 (149/385), but are much less frequent in the full-data-pretrained ResNet18 pipeline (14/385). This comparison shows that the supplied labels are substantially more separable under a different representation and training regime; it does not isolate whether pretraining, sample count, resolution or optimization is responsible. Median DenseNet121 softmax score on the wall-to-deck errors is 0.663. The explicit comparisons are reported in Table 13.

The distinction between the two tiers is therefore substantive: the full-data pipeline uses frozen pretrained features and a longer learned-head schedule, whereas the screen learns each entire network from random initialization under a short budget. The 10.54-percentage-point gap between their reported accuracies cannot be attributed solely to architecture. The following audits examine image dependence and training-budget sensitivity without reinterpreting surface-category recognition as damage assessment.

### 4.7. Content-Based Dependence Audit and Sensitivity

Table 14 reports the content-based dependence audit. The 17,086 inferred filename families do not cross the grouped partitions, but 9 byte-identical hash groups, 56 decoded-pixel-identical groups and 60 equal-difference-hash groups do. Combining these relations with near hashes at a Hamming distance of at most 3 produces 16,779 components; 69 components cross one or more original partitions. The largest component contains 193 images. Three combined components contain multiple supplied classes, which is a signal for provenance review rather than proof of annotation error. These cross-partition relations demonstrate that zero filename-family overlap does not establish a leakage-free split.

The content-screened subset retains 4102 of the 4202 original test images. Pretrained ResNet18 accuracy is 98.85% and macro-F1 is 0.9879; all 47 original errors remain, because the 100 excluded images were correctly classified by this pipeline. DenseNet121 retains 88.20% accuracy and 0.8749 macro-F1, and XCiT-Tiny retains 84.74% and 0.8406. These are post hoc subset estimates from fixed saved predictions. They show limited sensitivity to the specified exclusion rule, but the changed class composition and retained source dependence prevent us from treating the screened subset as a newly acquired independent test. All twenty screened results and component intervals are in Supplementary S1.

No independently acquired external field collection with verified parent-source exclusion and the same nine-category target definition is available for this study. Using a different portion of a contributing public dataset would not establish that independence. Remaining risks include overlapping source crops with different hashes, shared structures or acquisition conditions, and source-specific cues correlated with labels. A genuinely source/site-disjoint, prospectively held-out evaluation is therefore still required before making field-generalization claims.

An additional exploratory filename-prefix diagnostic reinforces that caution. After removing augmentation suffixes, numeric filenames of the form prefix–crop occur in 4044 deck images, 3829 wall images and 698 pavement images. All 54 deck prefixes and all 72 wall prefixes cross original partitions. Among test images matching this convention, all 404 decks and all 384 walls have a prefix also present outside testing; this also holds for 73 of 75 matching pavement test images. These prefixes are candidate source identifiers, not independently verified parent metadata. They are not merged into the reported content components, and the 4102-image screening result must not be interpreted as source-disjoint performance. Supplementary S1 supplies the complete prefix diagnostic. The illustrative error panel also avoids repeating a candidate prefix within each three-image row.

### 4.8. Training-Budget Sensitivity and Convergence Limits

**Figure 14:**
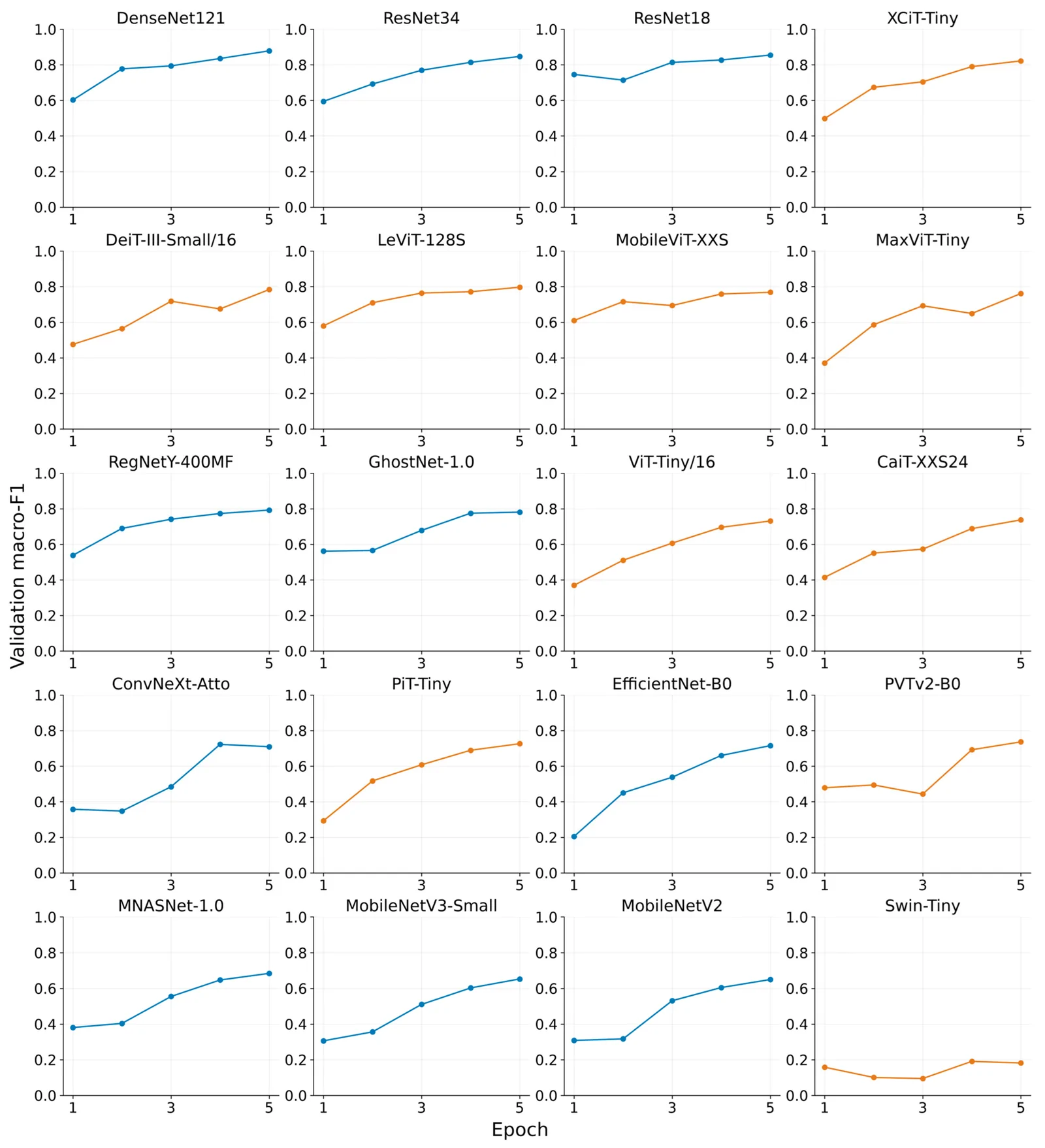
Recorded validation macro-F1 for all twenty original screening runs. Eighteen validation-selected checkpoints occur at epoch five; the remaining two occur at epoch four.

**Figure 15:**
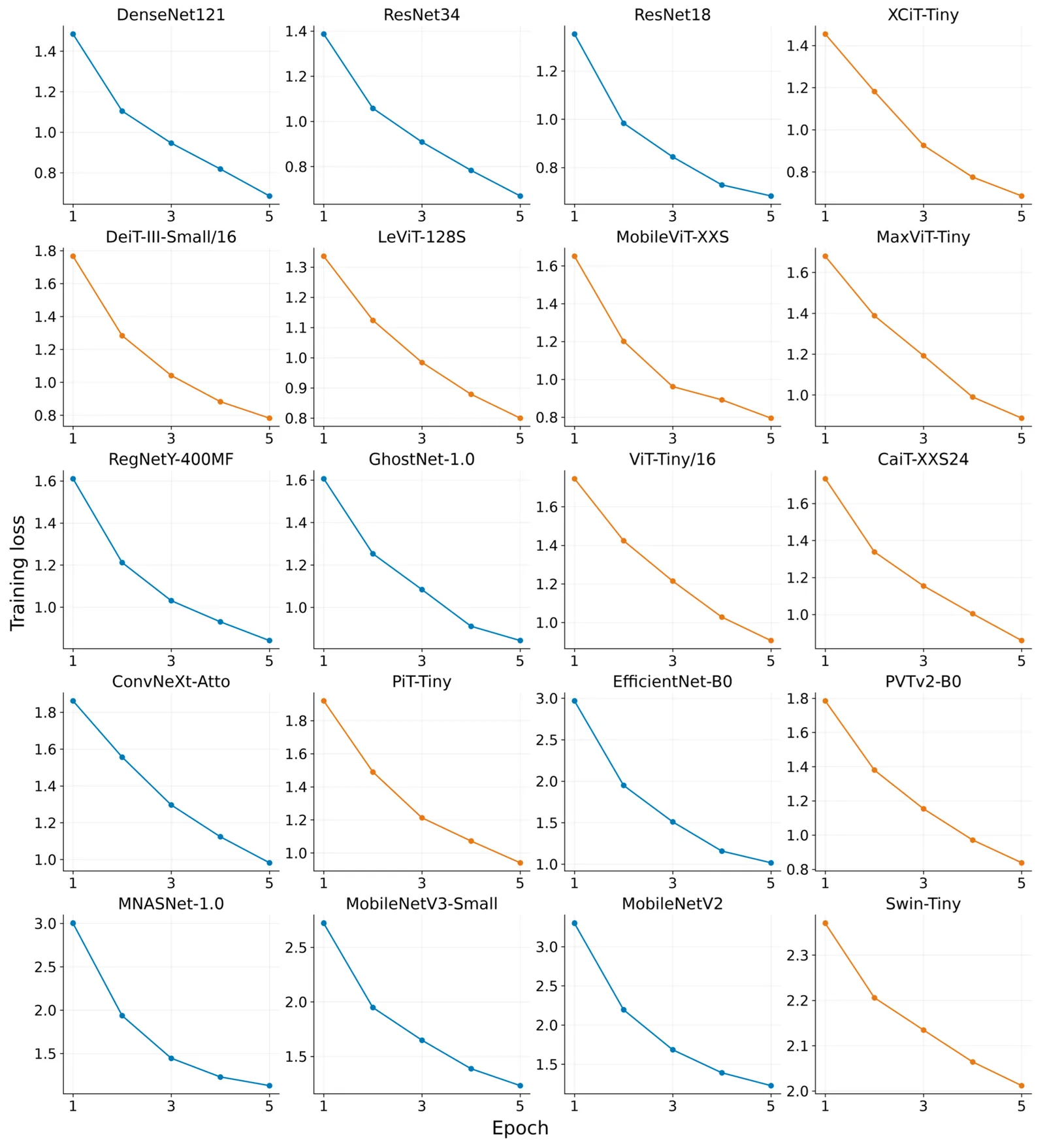
Recorded mean training loss for all twenty original screening runs. Declining loss and final-epoch validation improvements show why schedule completion cannot be equated with convergence.

Figure 14 and Figure 15 show every original architecture’s recorded validation macro-F1 and training loss. Eighteen of twenty models select epoch five and improve the validation macro-F1 between epochs four and five. DenseNet121 improves by 4.31 macro-F1 percentage points in that final epoch, XCiT-Tiny by 3.23, DeiT-III-Small/16 by 10.90 and MaxViT-Tiny by 11.23. ConvNeXt-Atto and Swin-Tiny select epoch four; that fact alone does not establish their convergence. The histories do not support the proposition that five epochs give all architectures comparable convergence. The results therefore characterize short-budget optimization rather than convergence-equivalent performance.

**Figure 16:**
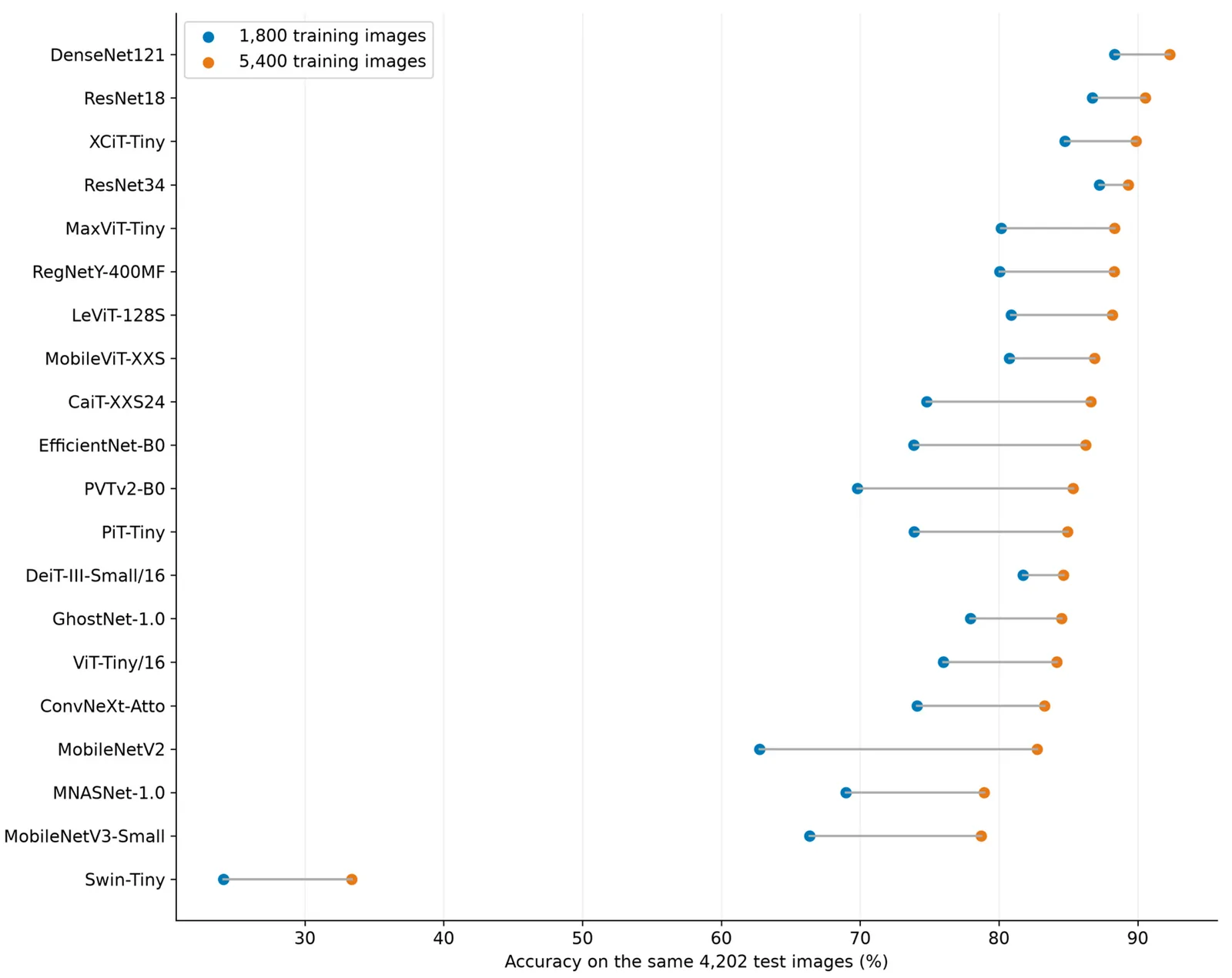
Training-data sensitivity at five epochs. Lines connect each architecture’s two measured accuracies; ordering follows the enlarged-data results.

Table 15 and Figure 16 report the completed 5400-image sensitivity runs for all twenty models. Relative to the original 1800-image screen, all twenty accuracies increase; changes range from 2.07 to 19.99 percentage points. Spearman rank correlation between the two sets of accuracies is 0.850. DenseNet121 has the highest observed test accuracy under both budgets. This comparison uses nested training samples and unchanged validation/test membership. It remains a single-seed, fixed-epoch sensitivity experiment, not proof of ranking stability at the full 33,352-image training size.

Increasing training data at fixed epoch count also increases gradient updates, so the result does not isolate data diversity from optimization exposure. The original unequal batch sizes remain a limitation, while the CPU thread setting differs for resource management. The same previously inspected test set is used only after validation-based checkpoint selection in each follow-up; this is a post hoc training-budget sensitivity analysis rather than external validation. Full-data, multi-seed, convergence-tuned comparisons and deployment-level timing remain outside the evidence reported here.

## 5. Conclusions

The study supports three conditional conclusions. First, an ImageNet-pretrained ResNet18 feature encoder with a modest learned head is a strong local baseline when the objective is organizing a large image collection into the same nine supplied surface categories: its original grouped accuracy is 98.88%, and its content-screened sensitivity accuracy is 98.85%. The remaining requirement is independent source/site validation, not a claim that numerical saturation has solved structural inspection. Second, DenseNet121 has the highest observed accuracy in the original five-epoch, 1800-image, 64 × 64 random-initialization screen. Eighteen final-epoch selections show that this is not a convergence-equivalent architecture comparison. The completed 5400-image sensitivity screen yields its highest observed accuracy for DenseNet121 (92.31%); all twenty models improve, with a between-budget rank correlation of 0.850. These changes combine more training images and more optimizer updates and do not establish full-data or converged rankings. Third, model choice depends on the operational constraint: the original ResNet18 screen offers lower measured CPU latency than DenseNet121 at a modest accuracy reduction; MobileNetV3-Small is faster but incurs a much larger classification loss; and MobileViT-XXS offers low parameter storage without correspondingly low CPU latency.

For engineering use, these findings justify selecting a candidate pipeline for a surface-image indexing stage and then validating it on the intended acquisition environment. They do not justify maintenance prioritization by damage severity, localization of cracks, assessment of deterioration mechanism or structural safety decisions. The data audit demonstrates why filename grouping, content similarity and source independence must be distinguished. Future validation should preserve known parent-image and site identifiers, use independent field collections, repeat training across seeds and budgets, and obtain the annotations required for any claimed damage-measurement task. Supplementary S1 documents the original runs and the dependence and training-budget analyses, enabling inspection of the reported computations and their scope.

## Figures and Tables

**Table 1:** Selected historical and methodological developments in structural-surface imagery, 2016–2026. Findings concern each study’s own task and are not directly comparable benchmark scores. The 2022 CrackSeg9k entry refers to its preprint release.

Year	Study	Task and Method	Finding	Relevance/Boundary
2016	Zhang et al. [1]	Pavement crack patches; CNN	Learned features for crack/background discrimination.	Binary crack recognition; not a nine-surface benchmark.
2017	Cha et al. [2]	Concrete cracks; CNN and sliding windows	About 98% patch accuracy; tested large images from a different structure.	Shows why separate-structure evidence matters.
2018	Dorafshan et al. [3]	SDNET2018; annotated deck, wall and pavement images	More than 56,000 patches from 230 parent images.	Parent-image dependence can persist after a random patch split.
2019	Zou et al. [4]	DeepCrack; multiscale encoder–decoder	Fuses hierarchical features to predict crack pixels.	Pixel localization requires masks absent from the present labels.
2020	Xiang et al. [5]	Pyramid context and spatial–channel attention	Combines context with local crack detail; evaluates three pavement datasets.	Attention was used before the recent transformer wave.
2021	Liu et al. [6]	CrackFormer; transformer encoder–decoder	Long-range attention and scaling attention for fine crack detection.	Segmentation evidence cannot be ranked against surface-label accuracy.
2022	Kulkarni et al. [11]	CrackSeg9k; consolidated crack segmentation benchmark	Harmonizes public image–mask data and compares segmentation approaches.	Shared annotations aid comparison; source lineage remains relevant.
2023	Tao et al. [7]	Convolutional-transformer and boundary awareness	Combines dilated local features, global context and boundary supervision.	Hybrid models benefit from task-specific supervision.
2024	Ye et al. [8]	CSW-S; residual cross-window transformer	Reports 96.92% binary crack classification on its own 17,000-image dataset.	Different labels, resolution and training schedule preclude a direct accuracy comparison.
2024	Benz and Rodehorst [12]	Omni-Crack30k; segmentation and transfer learning	Approximately 30,000 samples from more than twenty datasets.	Training representation and dataset composition affect comparability.
2025	Ye et al. [9]	transFissNet; CNN–transformer segmentation	Combines ResNet101 features and attention for concrete crack segmentation.	Surface-category labels cannot validate crack geometry.
2025	Liu et al. [13]	SCSegamba; structure-aware vision Mamba	Models crack morphology and texture with 2.8 M parameters.	Efficient pixel prediction; not part of the twenty-classifier screen.
2026	Ijaz et al. [10]	StructDamage; unified image classification release	Reports 98.62% DenseNet201 accuracy in the source study.	Release provenance and related-image partitioning must be made explicit.
2026	Asadi et al. [14]	CrackSegFlow; mask-conditioned image synthesis	Releases CSF-50K with 50,000 aligned image–mask pairs.	Synthetic segmentation supervision is not evaluated in this study.

**Table 2:** Class composition of the 41,756-image local balanced release used in the experiments.

Class	Images	Class	Images	Class	Images
Brick	4500	Cob	5000	Concrete	5280
Decks	4050	Pavements	4996	Road	4829
Stone	5000	Tile	4250	Walls	3851

**Table 3:** Partition protocols and their inferential roles. Zero filename-family overlap does not imply zero content or site overlap.

Protocol	Train	Validation	Test	Family Overlap	Analytical Role
Source-count-matched image-level	33,404	4148	4204	1941 crossing families	Sensitivity/comparability
Augmentation-family grouped	33,352	4202	4202	0	Original grouped estimate

**Table 4:** Implementation identifiers and resources for the original 20-model screen. Batch 12 or 32 is retained as recorded.

Family	Architecture	Implementation	Parameters (M)	Batch
CNN	ConvNeXt-Atto	convnext_atto	3.38	32
CNN	DenseNet121	densenet121	6.96	12
CNN	EfficientNet-B0	efficientnet_b0	4.02	32
CNN	GhostNet-1.0	ghostnet_100	3.91	32
CNN	MNASNet-1.0	mnasnet_100	3.11	32
CNN	MobileNetV2	mobilenetv2_100	2.24	32
CNN	MobileNetV3-Small	mobilenetv3_small_100	1.53	32
CNN	RegNetY-400MF	regnety_004	3.91	32
CNN	ResNet18	resnet18	11.18	32
CNN	ResNet34	resnet34	21.29	12
Transformer/attention	CaiT-XXS24	cait_xxs24_224	11.73	12
Transformer/attention	DeiT-III-Small/16	deit3_small_patch16_224	21.61	12
Transformer/attention	LeViT-128S	levit_128s	7.01	32
Transformer/attention	MaxViT-Tiny	maxvit_tiny_tf_224	30.38	12
Transformer/attention	MobileViT-XXS	mobilevit_xxs	0.95	32
Transformer/attention	PVTv2-B0	pvt_v2_b0	3.41	32
Transformer/attention	PiT-Tiny	pit_ti_224	4.55	32
Transformer/attention	Swin-Tiny	swin_tiny_patch4_window7_224	27.51	12
Transformer/attention	ViT-Tiny/16	vit_tiny_patch16_224	5.49	32
Transformer/attention	XCiT-Tiny	xcit_tiny_12_p16_224	6.53	32

**Table 5:** Operating principles of all twenty implementation variants. Source papers explain the architecture family; the present screen uses its own common training recipe.

Architecture	Working Mechanism
ConvNeXt-Atto [18]	Modern convolutional blocks with depthwise spatial filtering; Atto is the compact implementation variant.
DenseNet121 [19]	Concatenates earlier feature maps inside dense blocks to reuse features and propagate gradients.
EfficientNet-B0 [20]	Mobile inverted bottlenecks with squeeze–excitation; B0 is the baseline of compound scaling.
GhostNet-1.0 [21]	Uses inexpensive linear operations to produce additional feature maps from a smaller intrinsic set.
MNASNet-1.0 [22]	Mobile inverted bottlenecks from a latency-aware searched topology; no architecture search was rerun here.
MobileNetV2 [23]	Inverted residual blocks, depthwise convolutions and linear bottlenecks reduce computation.
MobileNetV3-Small [24]	Hardware-aware mobile blocks combine inverted residuals, squeeze–excitation and efficient activations.
RegNetY-400MF [25]	Regular stage widths and group convolutions; the Y variant includes squeeze–excitation.
ResNet18 [16]	Residual identity connections add block inputs to learned convolutional transformations; 18-layer configuration.
ResNet34 [16]	The same residual principle with more basic blocks; 34-layer configuration.
CaiT-XXS24 [26]	Deep token processing uses LayerScale, followed by class-attention aggregation.
DeiT-III-Small/16 [27]	ViT-style patch processing with LayerScale; the architecture is used, not the original DeiT III training recipe.
LeViT-128S [28]	A convolutional stem and progressively reduced token resolutions support efficient attention.
MaxViT-Tiny [29]	Mobile convolutional blocks combine local block attention with sparse global grid attention.
MobileViT-XXS [30]	Mobile convolutional blocks exchange local features with transformer-based global representations.
PVTv2-B0 [31]	Overlapping patch embedding, spatial-reduction attention and convolutional feed-forward layers form a pyramid.
PiT-Tiny [32]	Pooling between attention stages reduces spatial token dimensions while increasing representation width.
Swin-Tiny [33]	Hierarchical patch merging alternates regular and shifted-window attention.
ViT-Tiny/16 [34]	Non-overlapping 16 × 16 patches become tokens processed by global self-attention and a classifier token.
XCiT-Tiny [35]	Cross-covariance attention mixes feature channels, with local patch interaction and class-attention aggregation.

**Table 6:** Distinct purposes and budgets of the two original experimental tiers.

Element	Full-Data Primary Model	Controlled 20-Model Screen
Purpose	High-accuracy grouped classification	Matched architecture comparison
Initialization	ImageNet-pretrained ResNet18 encoder	Random for all 20 architectures
Training data	33,352 grouped images	1800 grouped images
Validation	4202 images	900 images
Test	4202 grouped images	4202 grouped images
Input	160 × 160 RGB crop	64 × 64 RGB
Selection	Best validation checkpoint	Best validation macro-F1

**Table 7:** Original full-data results with explicit protocol provenance. The source publication is contextual, not a paired comparator.

Model	Protocol	Test N	Accuracy	Macro-F1
Ijaz et al. DenseNet201 [10]	Reported image-level baseline	4204	98.6203%	0.98534
Local ResNet18	Source-count-matched image-level	4204	99.2388%	0.99160
Local ResNet18	Grouped primary	4202	98.8815%	0.98813

**Table 8:** Per-class performance of the original grouped pretrained ResNet18 pipeline.

Class	Precision	Recall	F1	Support
Brick	1.0000	0.9978	0.9989	450
Cob	1.0000	1.0000	1.0000	500
Concrete	0.9962	0.9886	0.9924	528
Decks	0.9522	0.9803	0.9660	406
Pavements	0.9704	0.9820	0.9761	500
Road	0.9979	0.9959	0.9969	483
Stone	0.9980	0.9880	0.9930	500
Tile	1.0000	1.0000	1.0000	450
Walls	0.9788	0.9610	0.9699	385

**Table 9:** Original 20-model screening results. The ranking is conditional on the short, low-resolution training protocol. T/A denotes transformer/attention.

Rank	Architecture	Family	Acc. (%)	Macro-F1	Params (M)	CPU ms	Pareto
1	DenseNet121	CNN	88.34	0.8765	6.96	65.53	Yes
2	ResNet34	CNN	87.24	0.8658	21.29	42.47	Yes
3	ResNet18	CNN	86.72	0.8612	11.18	15.51	Yes
4	XCiT-Tiny	T/A	84.75	0.8416	6.53	117.57	Yes
5	DeiT-III-Small/16	T/A	81.72	0.8135	21.61	45.13	
6	LeViT-128S	T/A	80.89	0.8039	7.01	45.69	Yes
7	MobileViT-XXS	T/A	80.75	0.8018	0.95	65.51	Yes
8	MaxViT-Tiny	T/A	80.15	0.7932	30.38	148.32	
9	RegNetY-400MF	CNN	80.06	0.7977	3.91	30.84	Yes
10	GhostNet-1.0	CNN	77.94	0.7776	3.91	77.29	
11	ViT-Tiny/16	T/A	75.99	0.7508	5.49	33.42	
12	CaiT-XXS24	T/A	74.80	0.7479	11.73	80.53	
13	ConvNeXt-Atto	CNN	74.11	0.7336	3.38	23.51	Yes
14	PiT-Tiny	T/A	73.89	0.7367	4.55	27.53	
15	EfficientNet-B0	CNN	73.87	0.7407	4.02	30.32	
16	PVTv2-B0	T/A	69.80	0.7012	3.41	69.10	
17	MNASNet-1.0	CNN	68.97	0.6956	3.11	42.98	Yes
18	MobileNetV3-Small	CNN	66.35	0.6653	1.53	10.42	Yes
19	MobileNetV2	CNN	62.76	0.6324	2.24	38.96	
20	Swin-Tiny	T/A	24.13	0.1642	27.51	111.76	

**Table 10:** Descriptive summaries of the ten selected architectures in each group under the original screening protocol. T/A denotes transformer/attention.

Family	n	Mean Acc. (%)	Median Acc. (%)	Mean Macro-F1	Mean Params (M)	Median CPU ms
CNN	10	76.63	76.02	0.7647	6.15	34.90
T/A	10	72.69	78.07	0.7155	11.92	67.31

**Table 11:** Exploratory paired DenseNet121–XCiT-Tiny accuracy contrast using component-bootstrap resampling.

Reference	Comparator	Δ Accuracy (pp)	95% Component CI (pp)	Resampling
DenseNet121	XCiT-Tiny	3.59	2.19 to 5.07	5000 component draws

**Table 12:** Paired component-bootstrap effects and secondary image-level McNemar/Holm diagnostics. Component intervals for ResNet34 and ResNet18 include zero.

Comparator	Δ (pp)	95% Component CI (pp)	Image Exact *p*	Image Holm *p*
ResNet34	1.09	−0.36 to 2.44	0.0079	0.0079
ResNet18	1.62	−0.07 to 3.28	0.000115	0.000229
XCiT-Tiny	3.59	2.19 to 5.07	2.22 × 10^−18^	6.66 × 10^−18^
DeiT-III-Small/16	6.62	3.19 to 10.10	3.02 × 10^−33^	1.21 × 10^−32^
LeViT-128S	7.45	4.87 to 10.06	2.19 × 10^−46^	1.09 × 10^−45^
MobileViT-XXS	7.59	5.19 to 10.15	1.68 × 10^−52^	1.35 × 10^−51^
MaxViT-Tiny	8.19	4.79 to 12.10	1.75 × 10^−52^	1.35 × 10^−51^
RegNetY-400MF	8.28	4.81 to 12.05	1.78 × 10^−49^	1.07 × 10^−48^
GhostNet-1.0	10.40	7.80 to 13.40	1.72 × 10^−83^	1.55 × 10^−82^
ViT-Tiny/16	12.35	8.42 to 16.34	7.16 × 10^−89^	7.16 × 10^−88^
CaiT-XXS24	13.54	8.24 to 19.06	2.21 × 10^−94^	2.43 × 10^−93^
ConvNeXt-Atto	14.23	10.60 to 18.25	1.16 × 10^−117^	1.62 × 10^−116^
PiT-Tiny	14.45	9.63 to 19.46	2.77 × 10^−112^	3.33 × 10^−111^
EfficientNet-B0	14.47	11.52 to 17.74	5.83 × 10^−114^	7.58 × 10^−113^
PVTv2-B0	18.54	13.45 to 23.75	2.67 × 10^−180^	4.27 × 10^−179^
MNASNet-1.0	19.37	15.09 to 23.64	8.76 × 10^−171^	1.31 × 10^−169^
MobileNetV3-Small	21.99	18.03 to 26.05	5.28 × 10^−221^	8.98 × 10^−220^
MobileNetV2	25.58	22.03 to 29.06	3.88 × 10^−228^	6.98 × 10^−227^
Swin-Tiny	64.21	59.62 to 68.19	<1 × 10^−300^	<1 × 10^−300^

**Table 13:** Wall/deck error asymmetry. Screening leaves all 385 wall images; original deck support is 406, and screened deck support is 404.

Model/Tier	Test Set	Wall → Deck	Wall Recall (%)	Deck → Wall
DenseNet121	Original	159/385 (41.30%)	58.18	5/406
DenseNet121	Screened	159/385 (41.30%)	58.18	5/404
XCiT-Tiny	Original	148/385 (38.44%)	59.74	6/406
XCiT-Tiny	Screened	148/385 (38.44%)	59.74	6/404
ResNet18	Original	149/385 (38.70%)	60.26	6/406
ResNet18	Screened	149/385 (38.70%)	60.26	6/404
Pretrained ResNet18	Original	14/385 (3.64%)	96.10	6/406
Pretrained ResNet18	Screened	14/385 (3.64%)	96.10	6/404

**Table 14:** Complete-image integrity audit. Counts refer to distinct relations and must not be added because their members can overlap.

Audit Relation	Distinct Groups/Components	Cross-Partition Groups	Interpretation
Inferred filename family	17,086	0	Suffix-derived family IDs only
Byte SHA-256	39,955	9	Identical stored file content
Decoded RGB + dimensions SHA-256	39,787	56	Identical decoded pixels
Equal 64-bit difference hash	35,781	60	Perceptual-hash equality; not proof of identity
Combined component; near-hash distance ≤ 3	16,779	69	Transitive filename/content/similarity links
Screened test subset	1661 test components	0 by the implemented links	4102 images; 100 original test images excluded

**Table 15:** All twenty original and enlarged-data runs. Accuracies use the same original 4202-image grouped test set; both budgets use five epochs.

Architecture	Original Rank	1800 Acc. (%)	5400 Acc. (%)	New Rank	Δ (pp)	New Val. Epoch
DenseNet121	1	88.34	92.31	1	3.97	5
ResNet18	3	86.72	90.55	2	3.83	5
XCiT-Tiny	4	84.75	89.89	3	5.14	5
ResNet34	2	87.24	89.31	4	2.07	5
MaxViT-Tiny	8	80.15	88.34	5	8.19	5
RegNetY-400MF	9	80.06	88.29	6	8.23	5
LeViT-128S	6	80.89	88.17	7	7.28	5
MobileViT-XXS	7	80.75	86.89	8	6.14	5
CaiT-XXS24	12	74.80	86.63	9	11.83	5
EfficientNet-B0	15	73.87	86.24	10	12.38	5
PVTv2-B0	16	69.80	85.34	11	15.54	5
PiT-Tiny	14	73.89	84.94	12	11.04	5
DeiT-III-Small/16	5	81.72	84.65	13	2.93	5
GhostNet-1.0	10	77.94	84.51	14	6.57	5
ViT-Tiny/16	11	75.99	84.17	15	8.19	5
ConvNeXt-Atto	13	74.11	83.29	16	9.19	5
MobileNetV2	19	62.76	82.75	17	19.99	5
MNASNet-1.0	17	68.97	78.94	18	9.97	5
MobileNetV3-Small	18	66.35	78.72	19	12.38	5
Swin-Tiny	20	24.13	33.37	20	9.23	5

## Data Availability

The raw data supporting the conclusions of this article will be made available by the authors on request.

## Supplementary Materials

The following supporting information can be downloaded at: https://www.mdpi.com/article/10.3390/jimaging12100487/s1, Supplementary S1: Methods, results and reproducibility evidence for Assessment of Convolutional Neural Network and Vision Transformer Architectures for Structural Surface Classification. Table S1: Class counts and grouped partition membership; Table S2: Effective training settings; Table S3: Implementation identifiers and optimizer exposure; Table S4: Full-data results and original image-bootstrap intervals; Table S5: Per-class performance of the grouped pretrained pipeline; Table S6: Complete original screen metrics; Table S7: Original resources and validation-selected checkpoints; Table S8: Complete enlarged screen metrics; Table S9: Original and enlarged data accuracy comparison; Table S10: Original validation macro F1 by epoch; Table S11: Enlarged validation macro F1 by epoch; Table S12: Distinct dependence relations across all 41,756 images; Table S13: Candidate numeric prefix overlap; Table S14: Original point estimates and 95 percent component intervals; Table S15: Screened point estimates and 95 percent component intervals; Table S16: Paired component effects and secondary exact McNemar results; Table S17: Observed directional errors in four recorded pipelines; Table S18: Image selection records for Figure S1; Table S19: Captured revision environment; Table S20: Reproducibility records in the embedded archive; Table S21: Key distributed file digests; Figure S1: Original DenseNet121 examples from the grouped test set.
