# Supplementary material for: Assessment of Convolutional Neural Network and Vision Transformer Architectures for Structural Surface Classification
Source: J Imaging. 2026 Oct 6;12(10):487. doi: 10.3390/jimaging12100487 (PMC13705699; doi:10.3390/jimaging12100487)
Supplement: Supplementary file 1 [file jimaging-12-00487-s001.zip › jimaging-4552724-supplementary.pdf]

Journal of
Imaging

MDPI

Supplementary Materials

Assessment of Convolutional Neural Network and Vision Transformer Architectures for Structural Surface Classification

Bonginkosi A. Thango [1,*] and Sipho G. Thango [2,3]

1 Department of Electrical and Electronic Engineering Technology, University of Johannesburg, Johannesburg 2092, South Africa
2 Discipline of Civil Engineering, University of KwaZulu Natal, Durban 4041, South Africa; thangos@ukzn.ac.za or siphot@nhbrc.org.za
3 National Home Builders Registration Council, Durban 4001, South Africa
* Correspondence: bonginkosit@uj.ac.za

This supplement accompanies the final manuscript and provides the experimental settings, complete model comparisons, image-dependence diagnostics, uncertainty estimates, training histories and reproducibility records. The results concern classification into nine supplied surface categories. They do not measure crack localization, damage severity, deterioration mechanisms or structural safety.

The full-data pretrained-feature pipeline and the two randomly initialized architecture screens are distinct experiments. Their principal findings are 98.88% grouped accuracy for the pretrained pipeline, 88.34% for DenseNet121 in the original screen and 92.31% for DenseNet121 in the enlarged-data screen. Content screening leaves the pretrained estimate at 98.85%; it does not establish source- or site-independent validation.

How to use this supplement

Sections S1–S3 define the data, training settings and model implementations. Sections S4–S6 report the full-data results, both architecture screens and their recorded histories. Sections S7–S9 provide dependence screening, component-aware comparisons and traceable error examples. Section S10 explains reproduction and contains the embedded evidence archive. Appendix A records file digests and the supplementary references.

The readable tables are accompanied by the complete machine-readable records and executable code embedded in this Word document. The archive preserves all 296 files of the verified computational supplement, including dataset-relative manifests and per-image predictions. Raw images, large model checkpoints and third-party pretrained weights are not redistributed; their availability and identity are distinguished in Section S10.

Reporting conventions: accuracy and accuracy intervals are percentages; accuracy differences are percentage points (pp). Precision, recall, F1, MCC and kappa are unitless proportions unless a table explicitly states otherwise. Rounded printed values do not replace the full-precision records. Supplementary table and figure numbers are separate from those in the main manuscript.

S1 Dataset identity and partition membership

The experiments use the 41,756-image local balanced derivative of StructDamage [1]. The nine folder labels mix materials and surface or element contexts. The separate 49,124-image release was not used for these experiments. No new field collection, pixel mask,

physical scale or severity label is introduced. Table S1 gives the exact class counts in the grouped partitions and in the content-screened test subset.

Table S1. Class counts and grouped partition membership.

Class	All images	Train	Validation	Test	Screened test
brick	4500	3600	450	450	450
cob	5000	4000	500	500	450
concrete	5280	4224	528	528	528
decks	4050	3238	406	406	404
pavements	4996	3996	500	500	462
road	4829	3863	483	483	473
stone	5000	4000	500	500	500
tile	4250	3350	450	450	450
walls	3851	3081	385	385	385
Total	41,756	33,352	4202	4202	4102

Filename family construction

The lower-case filename stem loses a terminal _aug followed by digits, optionally followed by _extra and digits, and then a terminal _extra followed by digits. Prefixing the normalized stem with its class forms the inferred augmentation-family identifier. Complete families are shuffled within each class with NumPy seed 20260923. Test and validation targets are each the rounded 10% class total; among targets not yet reached, the smaller allocated-count/target ratio is filled next, with test first on a tie. Remaining families enter training. Indivisible families can overshoot a target.

This produces 17,086 filename families with zero family overlap across the grouped partitions. It does not imply zero shared pixels or shared acquisition sources. The separate source-count-matched image-level split uses seed 20260822 and 33,404/4148/4204 images; 1941 inferred families cross those partitions. It matches reported counts, not the source publication's unknown exact assignments.

For the screens, the initial cache selects 600 training and 200 validation images per class from the grouped split with NumPy seed 20261024. The original screen uses the first 200 training and 100 validation cached members per class. The enlarged screen uses all 600 training members and the same 100 validation members. Both retain the same ordered 4,202-image test set. The effective manifests, not the larger cache manifest, define the experiments.

S2 Training settings and selection rules

The full-data encoder uses official ImageNet-pretrained ResNet18 weights [2,3] and remains frozen, including batch-normalization statistics. Table S2 distinguishes this feature pipeline from the randomly initialized screens. All runs are CPU-based; no supplementary result is an independent repetition across training seeds.

Table S2. Effective training settings.

Setting	Full-data feature pipeline	Original screen	Enlarged screen
Training / validation / test	33,352 / 4202 / 4202	1800 / 900 / 4202	5400 / 900 / 4202
Representation	Frozen 512-dimensional ResNet18 features	Entire model trained from random initialization	Entire model trained from random initialization
Input	Short side 176; center crop 160 × 160	RGB resize 64 × 64	RGB resize 64 × 64
Optimized parameters	Classifier head only	All model parameters	All model parameters
AdamW learning rate	0.002	0.0008	0.0008

https://doi.org/10.3390/jimaging12100487

Weight decay	0.0002	0.0005	0.0005
Cross-entropy label smoothing	0.03	0.05	0.05
Training batch	512	12 or 32	12 or 32
Epoch budget	100 maximum; early stopping	5	5
Cosine minimum learning rate	0.00002	0.00002	0.00002
Gradient clipping	Not applied in the saved head code	Norm 2.0	Norm 2.0
Training seed	20260923 for grouped head	20260822	20260822
Torch threads	Up to 10	10	4
Checkpoint selection	Validation macro-F1 improvement > 0.00001	Maximum validation macro-F1; earlier tie retained	Maximum validation macro-F1; earlier tie retained

The head is LayerNorm(512), Linear(512,384), GELU, dropout 0.15 and Linear(384,9). Features are stored as float16 and cast to float32 for optimization. The cosine scheduler spans 100 epochs. Early stopping follows 15 non-improving validation epochs after at least 25 epochs; the grouped run selects epoch 24 and finishes at epoch 39. The image-level head selects epoch 26 and finishes at epoch 41.

Screen training applies independent horizontal and vertical flips with probabilities 0.5 and 0.2. Per-image brightness and contrast factors are uniform on [0.9,1.1]; contrast is centered on each channel's spatial mean, then brightness is applied and values clipped to [0,1]. Channel normalization uses means (0.485,0.456,0.406) and standard deviations (0.229,0.224,0.225). Evaluation has no random augmentation. Incomplete training batches are dropped; evaluation batches retain all images.

S3 Architecture implementations and update budgets

Table S3 gives the exact timm identifiers and total parameter counts with nine-class outputs. Models are created with pretrained = False and num_classes = 9. The factory first passes img_size = 64; when a model rejects that argument with TypeError, it retries without the argument. All recorded inputs remain 64 × 64. T/A denotes transformer/attention, including hybrid implementations; names do not imply replication of the source papers' training recipes.

Table S3. Implementation identifiers and optimizer exposure.

Architecture	Group	timm identifier	Parameters	Batch	Updates 1800 / 5400
DenseNet121	CNN	densenet121	6,963,081	12	150 / 450
ResNet34	CNN	resnet34	21,289,289	12	150 / 450
ResNet18	CNN	resnet18	11,181,129	32	56 / 168
XCiT-Tiny	T/A	xcit_tiny_12_p16_224	6,525,009	32	56 / 168
DeiT-III-Small/16	T/A	deit3_small_patch16_224	21,608,841	12	150 / 450
LeViT-128S	T/A	levit_128s	7,009,098	32	56 / 168
MobileViT-XXS	T/A	mobilevit_xxs	953,913	32	56 / 168
MaxViT-Tiny	T/A	maxvit_tiny_tf_224	30,381,265	12	150 / 450
RegNetY-400MF	CNN	regnety_004	3,907,113	32	56 / 168
GhostNet-1.0	CNN	ghostnet_100	3,913,037	32	56 / 168
ViT-Tiny/16	T/A	vit_tiny_patch16_224	5,491,593	32	56 / 168
CaiT-XXS24	T/A	cait_xxs24_224	11,730,441	12	150 / 450
ConvNeXt-Atto	CNN	convnext_atto	3,377,409	32	56 / 168
PiT-Tiny	T/A	pit_ti_224	4,549,065	32	56 / 168

https://doi.org/10.3390/jimaging12100487

EfficientNet-B0	CNN	efficientnet_b0	4,019,077	32	56 / 168
PVTv2-B0	T/A	pvt_v2_b0	3,412,073	32	56 / 168
MNASNet-1.0	CNN	mnasnet_100	3,113,841	32	56 / 168
MobileNetV3-Small	CNN	mobilenetv3_small_100	1,527,081	32	56 / 168
MobileNetV2	CNN	mobilenetv2_100	2,235,401	32	56 / 168
Swin-Tiny	T/A	swin_tiny_patch4_window7_224	27,509,955	12	150 / 450

Updates are per epoch. Across five epochs the batch-12 models perform 750 versus 2250 updates, and the batch-32 models 280 versus 840. Equal epoch counts do not equalize optimizer updates or compute. The six batch-12 models are DenseNet121, ResNet34, Swin-Tiny, MaxViT-Tiny, DeiT-III-Small/16 and CaiT-XXS24.

The comparison spans residual and densely connected CNNs, mobile convolutions, modern convolutional blocks, patch attention, hierarchical attention and mobile hybrids. The models are deliberately selected implementations rather than a statistical sample of architectures. A 16 × 16 patch model begins with only 16 spatial tokens at this input size. These restrictions are part of the interpretation of the reported ranking.

S4 Full data pretrained feature results

Table S4 reports both original feature-pipeline protocols. Their test membership differs, so their accuracy difference is not a paired estimate of leakage inflation. Table S5 gives the nine-class profile for the grouped model. The grouped primary uncertainty interval is the component-bootstrap interval in Section S8, not the image-resampling interval retained here for provenance.

Table S4. Full-data results and original image-bootstrap intervals.

Metric	Image-level split	Filename-family grouped
Test images	4204	4202
Accuracy (%)	99.2388	98.8815
Macro precision	0.99152	0.98816
Macro recall / balanced accuracy	0.99171	0.98818
Macro F1	0.99160	0.98813
MCC	0.99143	0.98741
Cohen kappa	0.99143	0.98740
Image-bootstrap accuracy 95% interval (%)	98.98–99.50	98.55–99.19
Image-bootstrap macro-F1 95% interval	0.9886–0.9945	0.9846–0.9913
Best validation epoch	26	24

Original image-bootstrap intervals use 2000 resamples and treat stored images as independently resampled units. They are secondary sensitivity records, not the primary dependence-aware inference.

Table S5. Per-class performance of the grouped pretrained pipeline.

Class	Precision	Recall	F1	Support
brick	1.0000	0.9978	0.9989	450
cob	1.0000	1.0000	1.0000	500
concrete	0.9962	0.9886	0.9924	528
decks	0.9522	0.9803	0.9660	406
pavements	0.9704	0.9820	0.9761	500
road	0.9979	0.9959	0.9969	483
stone	0.9980	0.9880	0.9930	500
tile	1.0000	1.0000	1.0000	450
walls	0.9788	0.9610	0.9699	385

https://doi.org/10.3390/jimaging12100487

The grouped pipeline makes 47 errors among 4202 images. All 100 images removed by the content-screening rule were originally classified correctly, leaving the same 47 errors among 4,102 retained images. This fixed-prediction subset calculation explains the small accuracy change; it is not an external-validation experiment.

S5 Original architecture screen results

Table S6 contains every original screening result, ordered by observed test accuracy. Each architecture uses 1,800 training images, 900 validation images and the common 4,202-image test set. Checkpoints are validation-selected. Selecting the highest test-scoring architecture afterwards makes champion comparisons exploratory.

Table S6. Complete original screen metrics.

Architecture	Accuracy (%)	Macro precision	Macro recall	Macro F1	MCC	Kappa
DenseNet121	88.34	0.8907	0.8787	0.8765	0.8704	0.8687
ResNet34	87.24	0.8753	0.8690	0.8658	0.8578	0.8564
ResNet18	86.72	0.8745	0.8644	0.8612	0.8522	0.8505
XCiT-Tiny	84.75	0.8553	0.8457	0.8416	0.8302	0.8282
DeiT-III-Small/16	81.72	0.8320	0.8158	0.8135	0.7963	0.7941
LeViT-128S	80.89	0.8307	0.8080	0.8039	0.7881	0.7848
MobileViT-XXS	80.75	0.8238	0.8062	0.8018	0.7859	0.7832
MaxViT-Tiny	80.15	0.8086	0.8026	0.7932	0.7797	0.7766
RegNetY-400MF	80.06	0.8163	0.8006	0.7977	0.7775	0.7754
GhostNet-1.0	77.94	0.7990	0.7800	0.7776	0.7545	0.7516
ViT-Tiny/16	75.99	0.7973	0.7627	0.7508	0.7349	0.7297
CaiT-XXS24	74.80	0.7649	0.7500	0.7479	0.7183	0.7164
ConvNeXt-Atto	74.11	0.7471	0.7425	0.7336	0.7108	0.7085
PiT-Tiny	73.89	0.7579	0.7401	0.7367	0.7084	0.7061
EfficientNet-B0	73.87	0.7727	0.7420	0.7407	0.7103	0.7057
PVTv2-B0	69.80	0.7352	0.7011	0.7012	0.6633	0.6600
MNASNet-1.0	68.97	0.7275	0.6935	0.6956	0.6540	0.6502
MobileNetV3-Small	66.35	0.6934	0.6662	0.6653	0.6238	0.6208
MobileNetV2	62.76	0.6392	0.6344	0.6324	0.5818	0.5807
Swin-Tiny	24.13	0.1914	0.2499	0.1642	0.1843	0.1522

Balanced accuracy equals macro recall for these nine-class evaluations. All seven metrics, including this equality, were recomputed from the saved per-image true and predicted classes. No test score is substituted for the validation checkpoint-selection criterion.

DenseNet121 records the highest original accuracy, 88.34%, followed by ResNet34 and ResNet18. XCiT-Tiny leads the T/A group at 84.75%. These are short-budget observations, not converged performance ceilings. The selected CNNs have mean and median accuracies of 76.63% and 76.02%; the corresponding T/A summaries are 72.69% and 78.07%. Their different means and medians, and the low Swin-Tiny score, argue against a universal family-superiority interpretation.

S5 Original screen resources and selected epochs

Table S7 preserves the original timing and resource records. Batch-1 latency averages ten timed forward passes after three warm-ups and excludes image decoding. Recorded run time includes training, validation, checkpoint handling and test evaluation, but excludes cache preparation, model initialization and the latency loop. These host-specific timings are not pooled with the four-thread enlarged runs and do not establish end-to-end inspection throughput.

https://doi.org/10.3390/jimaging12100487

Table S7. Original resources and validation-selected checkpoints.

Architecture	Parameters (million)	Batch-1 CPU latency (ms)	Recorded run time (min)	Selected epoch	Observed Pareto set
DenseNet121	6.963	65.53	16.19	5	Yes
ResNet34	21.289	42.47	11.79	5	Yes
ResNet18	11.181	15.51	2.95	5	Yes
XCiT-Tiny	6.525	117.57	5.60	5	Yes
DeiT-III-Small/16	21.609	45.13	12.87	5	No
LeViT-128S	7.009	45.69	2.11	5	Yes
MobileViT-XXS	0.954	65.51	2.89	5	Yes
MaxViT-Tiny	30.381	148.32	35.69	5	No
RegNetY-400MF	3.907	30.84	3.27	5	Yes
GhostNet-1.0	3.913	77.29	4.03	5	No
ViT-Tiny/16	5.492	33.42	3.14	5	No
CaiT-XXS24	11.730	80.53	14.68	5	No
ConvNeXt-Atto	3.377	23.51	1.38	4	Yes
PiT-Tiny	4.549	27.53	2.62	5	No
EfficientNet-B0	4.019	30.32	3.36	5	No
PVTv2-B0	3.412	69.10	2.51	5	No
MNASNet-1.0	3.114	42.98	4.00	5	Yes
MobileNetV3-Small	1.527	10.42	1.26	5	Yes
MobileNetV2	2.235	38.96	2.19	5	No
Swin-Tiny	27.510	111.76	27.28	4	No

The non-dominated set jointly considers higher original accuracy, fewer parameters and lower measured latency. It is descriptive of these measurements. Spearman correlation is 0.438 between log10 parameter count and accuracy ($p = 0.054$), and 0.162 between latency and accuracy ($p = 0.494$). The original family median latencies are 34.90 ms for CNNs and 67.31 ms for T/A models.

ResNet18 is faster than DenseNet121 in this measurement but less accurate. MobileViT-XXS has about 0.95 million parameters yet a latency near 65.51 ms; small parameter storage therefore does not guarantee low CPU latency. No deployment-level energy, sustained throughput or field operating-cost study was performed.

S6 Enlarged architecture screen results

The enlarged screen restarts every architecture from random initialization with 5,400 training images, the unchanged 900-image validation sample and the same 4,202 test images. Table S8 gives all metrics for the validation-selected checkpoints in the archived macro-F1 order; accuracy ranks appear in Table S9. The budget remains five epochs and a single training seed.

Table S8. Complete enlarged screen metrics.

Architecture	Accuracy (%)	Macro precision	Macro recall	Macro F1	MCC	Kappa
DenseNet121	92.31	0.9316	0.9172	0.9164	0.9150	0.9134
ResNet18	90.55	0.9096	0.9004	0.8991	0.8948	0.8936
XCiT-Tiny	89.89	0.9063	0.8936	0.8921	0.8875	0.8861
ResNet34	89.31	0.9062	0.8875	0.8856	0.8818	0.8797
MaxViT-Tiny	88.34	0.8938	0.8792	0.8779	0.8703	0.8687
RegNetY-400MF	88.29	0.8900	0.8783	0.8770	0.8696	0.8681
LeViT-128S	88.17	0.8914	0.8777	0.8758	0.8685	0.8668
CaiT-XXS24	86.63	0.8676	0.8646	0.8636	0.8500	0.8494

https://doi.org/10.3390/jimaging12100487

MobileViT-XXS	86.89	0.8794	0.8648	0.8613	0.8543	0.8523
EfficientNet-B0	86.24	0.8774	0.8573	0.8552	0.8472	0.8450
PVTv2-B0	85.34	0.8523	0.8517	0.8503	0.8354	0.8350
PiT-Tiny	84.94	0.8602	0.8475	0.8451	0.8322	0.8304
DeiT-III-Small/16	84.65	0.8656	0.8433	0.8421	0.8300	0.8271
GhostNet-1.0	84.51	0.8528	0.8423	0.8394	0.8272	0.8255
ViT-Tiny/16	84.17	0.8531	0.8381	0.8380	0.8231	0.8217
ConvNeXt-Atto	83.29	0.8449	0.8313	0.8270	0.8143	0.8119
MobileNetV2	82.75	0.8420	0.8255	0.8220	0.8083	0.8057
MNASNet-1.0	78.94	0.8026	0.7891	0.7845	0.7654	0.7628
MobileNetV3-Small	78.72	0.7996	0.7869	0.7843	0.7623	0.7604
Swin-Tiny	33.37	0.2926	0.3371	0.2763	0.2630	0.2508

Balanced accuracy equals macro recall. Every enlarged run selected epoch five. Increasing training images at fixed epochs also increases gradient updates, so this is not a fixed-update experiment isolating data diversity. The previously inspected test set is reused only after validation selection in each run.

DenseNet121 again has the highest observed accuracy, 92.31%, followed by ResNet18 at 90.55% and XCiT-Tiny at 89.89%. The experiment does not train every architecture end to end on all 33,352 grouped training images, and it does not establish multi-seed or converged ranking stability.

S6 Measured changes between training budgets

Table S9 compares the nested budgets directly. All twenty accuracies improve, by 2.07–19.99 percentage points. Spearman rank correlation is 0.849624 (rounded to 0.850 in the manuscript). Only DenseNet121 and Swin-Tiny keep exactly the same rank. The comparison uses common test membership; it does not remove dependence among those test images.

Table S9. Original and enlarged data accuracy comparison.

Architecture	Original rank	1800 accuracy (%)	5400 accuracy (%)	Enlarged rank	Change (pp)
DenseNet121	1	88.34	92.31	1	3.97
ResNet18	3	86.72	90.55	2	3.83
XCiT-Tiny	4	84.75	89.89	3	5.14
ResNet34	2	87.24	89.31	4	2.07
MaxViT-Tiny	8	80.15	88.34	5	8.19
RegNetY-400MF	9	80.06	88.29	6	8.23
LeViT-128S	6	80.89	88.17	7	7.28
MobileViT-XXS	7	80.75	86.89	8	6.14
CaiT-XXS24	12	74.80	86.63	9	11.83
EfficientNet-B0	15	73.87	86.24	10	12.38
PVTv2-B0	16	69.80	85.34	11	15.54
PiT-Tiny	14	73.89	84.94	12	11.04
DeiT-III-Small/16	5	81.72	84.65	13	2.93
GhostNet-1.0	10	77.94	84.51	14	6.57
ViT-Tiny/16	11	75.99	84.17	15	8.19
ConvNeXt-Atto	13	74.11	83.29	16	9.19
MobileNetV2	19	62.76	82.75	17	19.99
MNASNet-1.0	17	68.97	78.94	18	9.97
MobileNetV3-Small	18	66.35	78.72	19	12.38
Swin-Tiny	20	24.13	33.37	20	9.23

https://doi.org/10.3390/jimaging12100487

The original unequal minibatches remain unchanged. Four Torch threads were requested for the enlarged runs instead of ten for the original screen to limit local contention. Consequently, time differences cannot be interpreted as the isolated computational cost of increasing dataset size. The saved results and execution log distinguish completed runs from interrupted attempts; only twenty completed five-epoch runs enter this table.

S6 Original validation histories

Table S10 reports all five recorded validation macro-F1 values as percentages for the original screen. These values determine checkpoint selection. Full per-epoch training loss, learning rate and validation metrics are retained in the embedded records; no curve is interpolated or presented as a separate training repetition.

Table S10. Original validation macro F1 by epoch.

Architecture	Epoch 1	Epoch 2	Epoch 3	Epoch 4	Epoch 5	Selected
DenseNet121	60.25	77.73	79.40	83.55	87.86	5
ResNet34	59.45	69.21	76.93	81.39	84.72	5
ResNet18	74.64	71.38	81.36	82.72	85.50	5
XCiT-Tiny	49.77	67.35	70.45	78.99	82.22	5
DeiT-III-Small/16	47.57	56.41	71.81	67.48	78.37	5
LeViT-128S	57.98	70.97	76.44	77.14	79.69	5
MobileViT-XXS	60.97	71.57	69.38	75.89	76.85	5
MaxViT-Tiny	37.10	58.61	69.27	64.92	76.14	5
RegNetY-400MF	53.83	68.94	74.12	77.33	79.25	5
GhostNet-1.0	56.14	56.56	67.83	77.51	78.10	5
ViT-Tiny/16	36.97	51.04	60.67	69.55	73.12	5
CaiT-XXS24	41.45	55.08	57.28	68.82	73.75	5
ConvNeXt-Atto	35.75	34.77	48.40	72.25	70.95	4
PiT-Tiny	29.29	51.72	60.78	68.94	72.64	5
EfficientNet-B0	20.45	45.00	53.78	66.01	71.54	5
PVTv2-B0	47.85	49.43	44.30	69.23	73.66	5
MNASNet-1.0	38.08	40.39	55.48	64.73	68.44	5
MobileNetV3-Small	30.62	35.63	51.05	60.28	65.31	5
MobileNetV2	30.84	31.69	53.10	60.48	64.99	5
Swin-Tiny	15.83	10.07	9.47	19.09	18.18	4

Eighteen models select epoch five and improve from epoch four to five. DenseNet121 gains 4.31 macro-F1 percentage points in that last epoch, XCiT-Tiny 3.23, DeiT-III-Small/16 10.90 and MaxViT-Tiny 11.23. ConvNeXt-Atto and Swin-Tiny select epoch four, which alone does not prove convergence. Five completed epochs therefore do not represent convergence-equivalent training.

S6 Enlarged validation histories

Table S11 reports all five recorded validation macro-F1 values as percentages for the enlarged screen. These values determine checkpoint selection. Full per-epoch training loss, learning rate and validation metrics are retained in the embedded records; no curve is interpolated or presented as a separate training repetition.

Table S11. Enlarged validation macro F1 by epoch.

Architecture	Epoch 1	Epoch 2	Epoch 3	Epoch 4	Epoch 5	Selected
DenseNet121	71.55	76.01	88.54	84.07	92.32	5
ResNet18	77.97	72.94	77.94	90.58	90.80	5
XCiT-Tiny	73.19	73.29	82.09	85.35	87.10	5
ResNet34	69.85	80.82	84.84	82.30	88.17	5
MaxViT-Tiny	69.14	76.51	75.79	85.95	89.00	5
RegNetY-400MF	72.26	76.18	81.95	86.44	86.48	5
LeViT-128S	76.00	74.29	86.15	81.40	87.48	5
CaiT-XXS24	61.43	68.84	80.83	82.92	85.46	5
MobileViT-XXS	75.46	80.80	83.56	83.56	84.59	5
EfficientNet-B0	47.16	75.71	70.03	83.05	84.11	5
PVTv2-B0	61.62	67.47	83.04	81.12	84.51	5
PiT-Tiny	61.44	70.43	74.49	82.16	86.14	5
DeiT-III-Small/16	60.92	74.40	76.15	78.75	81.22	5
GhostNet-1.0	69.97	80.94	82.56	84.08	85.44	5
ViT-Tiny/16	61.75	62.95	65.36	78.45	81.58	5
ConvNeXt-Atto	62.80	54.16	73.85	74.61	81.92	5
MobileNetV2	45.48	57.76	73.88	76.16	80.17	5
MNASNet-1.0	44.15	58.56	69.48	78.69	79.65	5
MobileNetV3-Small	40.89	67.57	68.94	76.22	78.42	5
Swin-Tiny	16.37	2.97	9.23	20.53	27.33	5

Every enlarged run selects the fifth and final epoch. The improved scores do not show where performance would plateau under longer training. No repeated-seed intervals or convergence-tuned comparisons are inferred from these histories.

S7 Content dependence audit

Table S12 summarizes the complete-image audit. Each stored file receives byte SHA-256, decoded-RGB-plus-dimensions SHA-256 and a 64-bit horizontal difference hash. The decoded-pixel digest hashes the UTF-8 encoding of Python's RGB size tuple followed by RGB pixel bytes. The perceptual hash resizes RGB to 9 × 8 using Pillow's default resize behavior, converts to grayscale, compares each right pixel with its left neighbor using strict greater-than, flattens in row-major order and assigns the first comparison to the least-significant bit.

Table S12. Distinct dependence relations across all 41756 images.

Relation	Groups or components	Repeated groups	Cross-partition groups	Cross-class groups
Filename family	17,086	3,430	0	0
Byte SHA-256	39,955	1,769	9	0
Decoded-pixel SHA-256	39,787	1,909	56	0
Equal difference hash	35,781	3,771	60	1
Combined component	16,779	Not applicable	69	3

Union-find components join equal filename families, equal byte hashes, equal decoded-pixel hashes and difference hashes with Hamming distance at most three, including cross-class matches. Four disjoint 16-bit blocks generate candidates; the full 64-bit distance confirms them. There are 9,902 distinct near-hash pairs at distance at most three. The largest combined component contains 193 images. Counts from different relations overlap and must not be added.

A test image is content-screened eligible only if its complete component contains no original training or validation image. The original test contains 1,703 components; the retained 4,102 images occupy 1,661 components. Exclusion uses content relationships rather than prediction correctness. Hash similarity can miss related crops or falsely join similar imagery; neither a component nor its isolation establishes a verified parent photograph or site.

Exploratory candidate source prefixes

Table S13 is a separate diagnostic. After augmentation-suffix removal, filenames matching two numeric groups separated by a hyphen receive the class-prefixed first number as a candidate identifier. These prefixes are not verified parents and are not added to the reported content components. The archive contains all 225 candidate-prefix group records and the full matching-image manifest.

Table S13. Candidate numeric prefix overlap.

Class	Matching images	Candidate prefixes	Prefixes crossing partitions	Matching test images	Test images with prefix elsewhere
decks	4044	54	54	404	404
pavements	698	99	70	75	73
walls	3829	72	72	384	384

S8 Component aware uncertainty on the original test

The primary uncertainty analysis extends bootstrap resampling [4] to the implemented dependence components [5]. It draws 1703 test components with replacement for each of 5000 replicates, seed 20260914. Every image in a sampled component is retained each time that component is selected. Thus replicate image counts can vary. Confusion matrices are summed with component multiplicities; 2.5th and 97.5th percentiles define the intervals in Table S14. The same component draws pair all model comparisons.

Table S14. Original point estimates and 95 percent component intervals.

Model	Accuracy (%) 95% interval	Macro F1 95% interval	Balanced accuracy (%) 95% interval
DenseNet121	88.34 [85.03–90.81]	0.8765 [0.8437–0.8985]	87.87 [84.81–90.16]
ResNet34	87.24 [83.88–89.66]	0.8658 [0.8320–0.8863]	86.90 [83.93–88.98]
ResNet18	86.72 [83.46–89.19]	0.8612 [0.8269–0.8831]	86.44 [83.36–88.76]
XCiT-Tiny	84.75 [81.40–87.32]	0.8416 [0.8056–0.8630]	84.57 [81.32–86.91]
DeiT-III-Small/16	81.72 [78.42–84.62]	0.8135 [0.7787–0.8377]	81.58 [78.51–84.42]
LeViT-128S	80.89 [77.58–83.75]	0.8039 [0.7707–0.8267]	80.80 [78.01–83.21]
MobileViT-XXS	80.75 [77.08–83.97]	0.8018 [0.7636–0.8286]	80.62 [77.18–83.66]
MaxViT-Tiny	80.15 [75.94–83.81]	0.7932 [0.7506–0.8240]	80.26 [76.43–83.67]
RegNetY-400MF	80.06 [76.31–83.42]	0.7977 [0.7605–0.8258]	80.06 [76.77–83.28]
GhostNet-1.0	77.94 [73.80–81.42]	0.7776 [0.7327–0.8076]	78.00 [73.87–81.47]
ViT-Tiny/16	75.99 [72.31–79.34]	0.7508 [0.7157–0.7771]	76.27 [73.03–79.29]
CaiT-XXS24	74.80 [69.44–79.87]	0.7479 [0.6966–0.7910]	75.00 [70.25–79.78]
ConvNeXt-Atto	74.11 [69.84–78.05]	0.7336 [0.6909–0.7665]	74.25 [70.55–77.82]
PiT-Tiny	73.89 [69.14–78.42]	0.7367 [0.6880–0.7760]	74.01 [69.40–78.39]
EfficientNet-B0	73.87 [70.11–77.23]	0.7407 [0.6987–0.7673]	74.20 [70.57–77.35]
PVTv2-B0	69.80 [64.18–75.39]	0.7012 [0.6430–0.7488]	70.11 [64.66–75.59]
MNASNet-1.0	68.97 [64.64–73.00]	0.6956 [0.6499–0.7283]	69.35 [65.12–73.30]

https://doi.org/10.3390/jimaging12100487

MobileNetV3-Small	66.35 [61.96–70.57]	0.6653 [0.6151–0.7007]	66.62 [62.11–70.85]
MobileNetV2	62.76 [58.83–66.52]	0.6324 [0.5890–0.6617]	63.44 [59.47–66.98]
Swin-Tiny	24.13 [21.36–27.20]	0.1642 [0.1462–0.1827]	24.99 [23.74–26.30]
Pretrained ResNet18	98.88 [98.42–99.26]	0.9881 [0.9837–0.9919]	98.82 [98.37–99.20]

The final row is the separately trained full-data feature pipeline, not a twenty-first model in the random-initialization screen. Intervals condition on the fixed recorded models and operational components. They do not include training-seed uncertainty or uncertainty about missing parent/site metadata. Zero-denominator class precision and F1 are zero; macro metrics use all nine class positions.

S8 Content screened results for every model

Table S15 gives the fixed-prediction estimates on the 4,102 retained images and their 95% component-bootstrap intervals. It resamples the 1,661 retained components 2,000 times with seed 20260914. No model is retrained for this screening analysis, and no new test images are acquired.

Table S15. Screened point estimates and 95 percent component intervals.

Model	Accuracy (%) 95% interval	Macro F1 95% interval	Balanced accuracy (%) 95% interval
DenseNet121	88.20 [85.02–90.66]	0.8749 [0.8417–0.8969]	87.64 [84.46–90.04]
ResNet34	87.18 [83.97–89.68]	0.8645 [0.8308–0.8860]	86.67 [83.59–88.96]
ResNet18	86.59 [83.50–89.07]	0.8592 [0.8252–0.8810]	86.17 [83.08–88.59]
XCiT-Tiny	84.74 [81.57–87.28]	0.8406 [0.8061–0.8631]	84.41 [81.25–86.87]
DeiT-III-Small/16	81.69 [78.07–84.65]	0.8122 [0.7738–0.8357]	81.46 [78.25–84.14]
LeViT-128S	80.91 [77.66–83.80]	0.8022 [0.7680–0.8258]	80.60 [77.77–83.25]
MobileViT-XXS	81.42 [77.80–84.50]	0.8070 [0.7674–0.8329]	81.06 [77.44–83.97]
MaxViT-Tiny	80.40 [76.11–84.10]	0.7933 [0.7491–0.8260]	80.18 [76.13–83.63]
RegNetY-400MF	79.91 [76.03–83.27]	0.7940 [0.7545–0.8213]	79.66 [76.09–82.90]
GhostNet-1.0	77.99 [74.10–81.45]	0.7769 [0.7329–0.8071]	77.92 [73.67–81.38]
ViT-Tiny/16	76.16 [72.39–79.64]	0.7492 [0.7118–0.7752]	75.98 [72.39–79.02]
CaiT-XXS24	74.57 [69.37–79.74]	0.7425 [0.6899–0.7862]	74.45 [69.40–79.23]
ConvNeXt-Atto	74.06 [69.76–77.93]	0.7295 [0.6847–0.7620]	73.72 [69.90–77.13]
PiT-Tiny	73.70 [68.97–78.46]	0.7317 [0.6812–0.7715]	73.44 [68.60–77.98]
EfficientNet-B0	74.13 [69.98–77.61]	0.7417 [0.6993–0.7686]	74.36 [70.60–77.57]
PVTv2-B0	69.43 [63.94–74.74]	0.6970 [0.6390–0.7440]	69.65 [64.15–74.99]
MNASNet-1.0	69.62 [65.58–73.72]	0.6991 [0.6529–0.7309]	69.66 [65.30–73.59]
MobileNetV3-Small	67.28 [63.08–71.31]	0.6720 [0.6214–0.7071]	67.30 [62.65–71.40]
MobileNetV2	62.97 [59.01–66.76]	0.6327 [0.5888–0.6630]	63.42 [59.41–67.12]
Swin-Tiny	24.31 [21.65–27.48]	0.1650 [0.1463–0.1839]	24.95 [23.70–26.24]
Pretrained ResNet18	98.85 [98.38–99.23]	0.9879 [0.9832–0.9917]	98.80 [98.35–99.19]

The retained class totals are in Table S1. Exclusions alter class composition. The pretrained pipeline remains at 98.85% accuracy, DenseNet121 at 88.20% and XCiT-Tiny at 84.74%. This sensitivity to implemented content links cannot establish independent-source or independent-site generalization.

S8 Paired effects and secondary image diagnostics

Table S16 contrasts original DenseNet121 accuracy with each alternative on common test images. The effect interval resamples identical components for both models. Intervals against ResNet34 and ResNet18 include zero; the interval against XCiT-Tiny is 2.19–5.07 pp. These are exploratory contrasts after inspecting test scores, not confirmatory tests of architecture-family superiority.

Table S16. Paired component effects and secondary exact McNemar results.

Comparator	Difference (pp)	Component 95% interval (pp)	Dense only / other only correct	Exact image p	Holm image p
ResNet34	1.09	-0.36 to 2.44	167 / 121	0.0079	0.0079
ResNet18	1.62	-0.07 to 3.28	186 / 118	1.15e-04	2.29e-04
XCiT-Tiny	3.59	2.19 to 5.07	229 / 78	2.22e-18	6.66e-18
DeiT-III-Small/16	6.62	3.19 to 10.10	417 / 139	3.02e-33	1.21e-32
LeViT-128S	7.45	4.87 to 10.06	412 / 99	2.19e-46	1.09e-45
MobileViT-XXS	7.59	5.19 to 10.15	397 / 78	1.68e-52	1.35e-51
MaxViT-Tiny	8.19	4.79 to 12.10	445 / 101	1.75e-52	1.35e-51
RegNetY-400MF	8.28	4.81 to 12.05	468 / 120	1.78e-49	1.07e-48
GhostNet-1.0	10.40	7.80 to 13.40	505 / 68	1.72e-83	1.55e-82
ViT-Tiny/16	12.35	8.42 to 16.34	630 / 111	7.16e-89	7.16e-88
CaiT-XXS24	13.54	8.24 to 19.06	701 / 132	2.21e-94	2.43e-93
ConvNeXt-Atto	14.23	10.60 to 18.25	681 / 83	1.16e-117	1.62e-116
PiT-Tiny	14.45	9.63 to 19.46	710 / 103	2.77e-112	3.33e-111
EfficientNet-B0	14.47	11.52 to 17.74	707 / 99	5.83e-114	7.58e-113
PVTv2-B0	18.54	13.45 to 23.75	833 / 54	2.67e-180	4.27e-179
MNASNet-1.0	19.37	15.09 to 23.64	902 / 88	8.76e-171	1.31e-169
MobileNetV3-Small	21.99	18.03 to 26.05	977 / 53	5.28e-221	8.98e-220
MobileNetV2	25.58	22.03 to 29.06	1185 / 110	3.88e-228	6.98e-227
Swin-Tiny	64.21	59.62 to 68.19	2727 / 29	<1e−300	<1e−300

Image-level Cochran Q is 13,299.10065 with 19 degrees of freedom; the computed tail probability underflows to zero. Exact two-sided image McNemar tests use the discordant counts in Table S16, with Holm adjustment across nineteen contrasts. All adjusted values are below 0.05, but clustering and test-based champion selection prevent treating these as independent confirmatory significance declarations. Holm correction does not repair those problems.

The archive also retains component_bootstrap_sign_tail_p as a descriptive bootstrap sign-tail diagnostic. It is not a null-calibrated permutation p-value and is not substituted for the paired effect intervals.

S9 Wall and deck error structure

Table S17 quantifies the original-versus-screened wall/deck asymmetry. DenseNet121 maps 159 of 385 walls to decks, accounting for 159 of its 490 original errors (32.45%). Only 5 of 406 decks are mapped to walls. Content screening retains all 385 walls and 404 decks, leaving the same 159 wall-to-deck errors.

Table S17. Observed directional errors in four recorded pipelines.

Model	Test subset	Wall to deck	Wall recall (%)	Deck to wall
DenseNet121	Original	159/385	58.18	5/406
DenseNet121	Screened	159/385	58.18	5/404
XCiT-Tiny	Original	148/385	59.74	6/406
XCiT-Tiny	Screened	148/385	59.74	6/404
ResNet18	Original	149/385	60.26	6/406
ResNet18	Screened	149/385	60.26	6/404
Pretrained ResNet18	Original	14/385	96.10	6/406
Pretrained ResNet18	Screened	14/385	96.10	6/404

Worked wall calculation

For original DenseNet121, the two-by-two matrix has rows [wall, not wall] and columns [predicted wall, predicted not wall]: first row [224,161], second row [22,3795]. Wall precision is 224/246 = 0.9106, recall is 224/385 = 0.5818 and F1 is 448/631 = 0.7100. Of the 161 false negatives, 159 are predicted decks and the remaining two concrete and stone. This is an observed result, not a synthetic example.

The median maximum-softmax score among DenseNet121's wall-to-deck errors is 0.663. Maximum softmax is not calibrated confidence or a probability of structural damage. The full-data pretrained pipeline has fewer wall-to-deck errors, but pretraining, input resolution, training-set size and optimization all differ, so this comparison does not isolate an architectural cause.

Traceable examples

Figure S1 reproduces the nine actual test photographs used in the manuscript's Figure 13. Table S18 identifies every image. Within each row, examples are selected in decreasing maximum-softmax order while avoiding repeated audit components and candidate numeric prefixes. The selection is post hoc and illustrative, not a random sample. Local texture or missing scene context is a plausible visual explanation, not evidence of an annotation error or a controlled mechanism test.

S9 Actual image examples

The three rows in Figure S1 show wall-to-deck errors, correctly classified walls and correctly classified decks, respectively. Images retain their original content and black figure lettering.

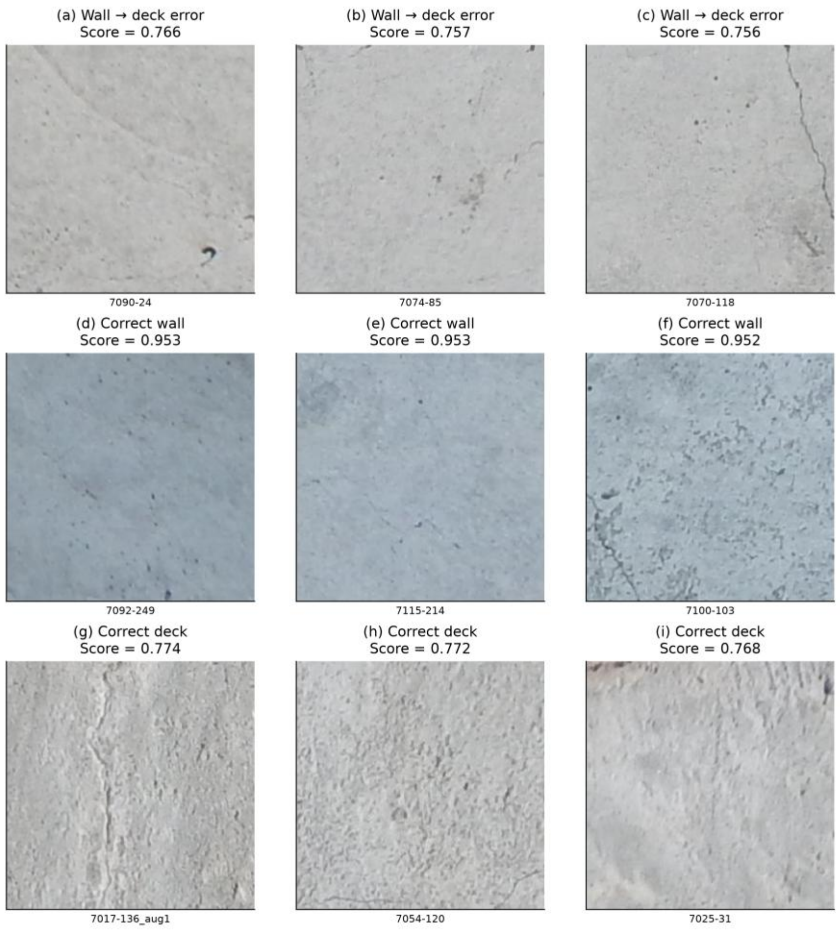

Figure S1. Original DenseNet121 examples from the grouped test set. Panels (a–c) are wall-to-deck

errors, (d–f) correct walls and (g–i) correct decks. Displayed scores are uncalibrated maximum softmax outputs. Table S18 supplies dataset-relative filenames and dependence identifiers.

S9 Example image traceability

Table S18 records the exact images in Figure S1 in reading order. Paths are relative to the directory containing the nine class folders. The full wall_panel_selection.csv includes numeric labels, correctness, confidence, component, candidate prefix and selection group.

Table S18. Image selection records for Figure S1.

Panel	Dataset-relative image	True / predicted	Score	Component	Candidate prefix
a	walls/7090-24.jpg	walls / decks	0.765918	c032472	walls::7090
b	walls/7074-85.jpg	walls / decks	0.756661	c041602	walls::7074
c	walls/7070-118.jpg	walls / decks	0.755526	c041461	walls::7070
d	walls/7092-249.jpg	walls / walls	0.952630	c037178	walls::7092
e	walls/7115-214.jpg	walls / walls	0.952523	c031054	walls::7115
f	walls/7100-103.jpg	walls / walls	0.951844	c033070	walls::7100
g	decks/7017-136_aug1.jpg	decks / decks	0.774011	c014623	decks::7017
h	decks/7054-120.jpg	decks / decks	0.772038	c012366	decks::7054
i	decks/7025-31.jpg	decks / decks	0.768306	c012122	decks::7025

The examples do not establish verified parent-image independence. The candidate prefix check adds diversity to each displayed row, but the prefix is not independently confirmed acquisition metadata. No expert relabeling, crack-mask evaluation or background-removal ablation was performed.

Metric computation and numerical conventions

Confusion matrices use true classes as rows and predictions as columns, in the fixed order brick, cob, concrete, decks, pavements, road, stone, tile, walls. Accuracy is the diagonal total divided by image count. Per-class precision divides the diagonal by predicted-class count; recall divides it by true-class count. Per-class F1 is twice the diagonal divided by the sum of true and predicted counts. Macro scores average the nine class scores; balanced accuracy equals macro recall. Zero denominators contribute zero.

MCC uses the multiclass confusion matrix, while Cohen kappa adjusts observed agreement for the agreement implied by true and predicted class marginals. Printed scores round the full-precision values. The saved prediction files retain the supplied and predicted label, category name, maximum-softmax score and correctness flag for every image. Raw class-probability vectors are not implied where only a maximum score was saved.

S10 Software and offline reproduction

Table S19 records the environment captured for the audit and enlarged-data analyses. It is not a claim that the entire original training environment was independently archived. The host was an Intel Core i5-1235U CPU with 12 logical processors on Windows; no GPU was used. Software builds, thread scheduling and floating-point behavior can affect reruns.

Table S19. Captured revision environment.

Component	Version
Python	3.11.9
Operating system	Windows-10-10.0.26200-SP0

torch	2.5.1+cpu
torchvision	0.20.1+cpu
timm	1.0.28
numpy	2.4.6
pandas	3.0.5
scipy	1.17.1
scikit-learn	1.9.0
Pillow	12.3.0
matplotlib	3.11.1
seaborn	0.13.2

Reproduction sequence

Save and extract the embedded archive in Section S10 below. Before offline use, obtain the exact balanced image files and required libraries. Install the versions in requirements-revision.txt; CPU PyTorch wheels may require the official wheel distribution. Raw images remain available from the authors on request, as stated in the manuscript, subject to the originating datasets' terms. The scripts do not download data or weights.

Start with prediction verification, which needs no images or retraining. The hashes command checks the byte identity of all 41,756 images once the data root is supplied. Analyze recomputes uncertainty, contrasts and budget comparison from saved predictions. Figures uses the images for the illustrative panel. Training commands require a new output directory so archived results are not overwritten.

```
python reproduce.py validate
python reproduce.py hashes --data-root "D:/data/Balanced/Balanced"
python reproduce.py analyze
python reproduce.py figures --data-root "D:/data/Balanced/Balanced"
```

S10 Training commands and embedded evidence

Run commands from the extracted archive root. The example paths identify local data and new output directories; substitute the actual locations. Screen commands run all twenty architectures by default; adding --model ResNet18 performs a single-model check. All screen reruns use the saved effective membership, not a newly sampled partition.

```
python reproduce.py screen-original --data-root "D:/data/Balanced/Balanced" --output "D:/runs/original-screen"

python reproduce.py screen-expanded --data-root "D:/data/Balanced/Balanced" --output "D:/runs/expanded-screen"

python reproduce.py full-grouped --data-root "D:/data/Balanced/Balanced" --weights "D:/weights/resnet18-f37072fd.pth" --output "D:/runs/full-grouped"
```

The grouped command reconstructs the frozen features using the recorded short-side resize of 176 and 160 × 160 center crop. It checks the official weight digest before training. It does not replace this transform with a standard 224 × 224 weight transform. Large learned checkpoints and feature caches are excluded from the archive, but checkpoint digests are retained.

Embedded computational supplement

The object below contains the complete verified reproducibility ZIP inside this Word document. In desktop Microsoft Word, open or save the embedded package, then extract the ZIP to a writable directory. Embedded-object activation can be restricted by a

document viewer or organizational security policy; the readable methods and results remain available without activating it. No macro or code runs automatically when this document is opened.

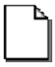

Embedded archive: jimaging-4552724_Supplementary_S1.zip
296 files; 23,514,120 bytes.
Archive SHA-256
efcf78d1798ab0f0dfc4fb0df9dbf284be8fa0b2d99a0de70bfba393bd2ced2d

The byte-identical archive separates original experiments from post-review sensitivity analyses. CHECKSUMS.sha256 covers the other 295 members; provenance.json preserves original and distributed digests and identifies portable-path normalization. The execution log includes interrupted attempts and their continuation. Completed metrics, predictions and five-epoch histories alone contribute to the twenty enlarged results.

S10 Evidence organization and file schemas

Table S20 maps the readable supplement to its embedded records. P1 denotes Paper1_DL_Classification_Pack and JR denotes JournalImaging_4552724_Revision within the extracted archive. These are retained provenance directory names; they do not alter the identity of manuscript jimaging-4552724.

Table S20. Reproducibility records in the embedded archive.

Evidence	Archive location or naming rule
Original source code	P1/run_classification.py; run_grouped_head.py; run_20_model_benchmark.py; finalize_results.py; finalize_20_model_results.py
Full-data manifests and predictions	P1/results/split_manifest.csv; grouped_split_manifest.csv; test_predictions_resnet18*.csv
Original screen outputs	P1/results/benchmark20/: metrics_*, predictions_*, history_* and Table_20_model_results.csv
Effective screen membership	JR/results/original_screen_actual_manifest.csv; expanded/actual_split_manifest.csv
Enlarged screen and run settings	JR/results/expanded/: all twenty metrics, predictions and histories; protocol.json; execution.log
Content and filename audit	JR/results/image_integrity.csv; audited_manifest.csv; audit_components.csv; near_hash_pairs.csv
Intervals and contrasts	JR/results/component_confidence_intervals.csv; screened_component_intervals.csv; component_paired_contrasts.csv; image_mcnemar_holm.csv
Candidate source prefixes	JR/results/candidate_source_prefix_manifest.csv; candidate_source_prefix_groups.csv; candidate_source_prefix_summary.csv
Error panel and budget comparison	JR/results/wall_panel_selection.csv; wall_deck_diagnostic.csv; data_budget_comparison.csv; convergence_audit.csv
Analysis and figure code	JR/audit_compute.py; analyze_results.py; source_prefix_audit.py; verify_computations.py; make_figures.py; run_followup.py
Figure sources	JR/figures/ (PNG and SVG); retained original image panels in JR/audit/original_media/

Entry point and provenance	reproduce.py; README.md; requirements-revision.txt; checkpoint_digests.json; provenance.json; CHECKSUMS.sha256

Manifest records identify path, class_name, numeric label, split and augmentation_family. The audited manifest adds sha256, pixel_sha256, dhash, width, height, audit_component and strict_test_eligible. Prediction files contain path, true/predicted labels and classes, confidence and correctness. Histories contain epoch, training loss, learning rate and validation metrics. Component-interval records contain model, metric, estimate, bounds, component count and replicate count.

Machine-readable records, rather than printed rounding, are the reproduction targets. Recomputing from saved predictions does not establish independent field validation. The documented limitations on target labels, source dependence, limited training budgets and single-seed inference continue to apply.

Appendix A File identity and supplementary references

Table S21 records SHA-256 digests of key distributed manifests and outputs. These hashes describe the portable files inside the embedded archive, not the original absolute-path versions. The complete per-file ledger and source-to-distributed mapping remain in CHECKSUMS.sha256 and provenance.json.

Table S21. Key distributed file digests.

Archive member	SHA-256
P1/results/grouped_split_manifest.csv	ddcfaa361cecc7b535c1fdff43f94c8f 12fd6aaa6ab284e5dceed5ef23a22d31
P1/results/split_manifest.csv	1325caa06696d1906a1cbb3960601bf2 5c319b517520b15ce3759ba63823776b
P1/results/test_predictions_resnet18_grouped.csv	319eafa804814cd3ac85df281ae11563 605dc056995425ccfd87ed459aa5308d
JR/results/audited_manifest.csv	caa928b7c19a51239d19a059ad2551f6 5e47915c7ea5f75d8c5a54eb343da571
JR/results/data_budget_comparison.csv	09fb4afead448d3a3b0aaa2c9eb20b04 e4a00aab7c065be335b6c1d445f7cb5f
JR/results/expanded/actual_split_manifest.csv	59c2a1b74c5980d000740b62206321b5 15261d0d488a73a08cb7e1710dfc4929
JR/results/image_integrity.csv	043e3254c18b0f59d9571127cb7f5ee5 ca30b55d86c53bac1b98a63b29ab495a
JR/results/original_screen_actual_manifest.csv	4d3312abbdc7f650b4af96604ffda41d 4efd48988b821a3119f78a3820acac4f
CHECKSUMS.sha256	08a0ad9b1c6c98188c1f63ea6fbca9ad ad899c89a6aef903ac2f76f327f66f62

Pretrained encoder identity

Official weight file: resnet18-f37072fd.pth. Its SHA-256 digest is:
f37072fd47e89c5e827621c5baffa7500819f7896bbacec160b1a16c560e07ec

Supplementary references

1. Ijaz, M.; Khan, S.U.R.; Rehman, A.U.; Vollmer, S.; Dengel, A.; Asim, M.N. StructDamage: A large scale unified crack and surface defect dataset for robust structural damage detection. arXiv 2026, arXiv:2603.10484v1. https://doi.org/10.48550/arXiv.2603.10484.
2. Deng, J.; Dong, W.; Socher, R.; Li, L.-J.; Li, K.; Fei-Fei, L. ImageNet: A large-scale hierarchical image database. In Proceedings of the 2009 IEEE Conference on Computer Vision and Pattern Recognition, Miami, FL, USA, 20–25 June 2009; pp. 248–255. https://doi.org/10.1109/CVPR.2009.5206848.

3. He, K.; Zhang, X.; Ren, S.; Sun, J. Deep residual learning for image recognition. In Proceedings of the IEEE Conference on Computer Vision and Pattern Recognition, Las Vegas, NV, USA, 27–30 June 2016; pp. 770–778. https://doi.org/10.1109/CVPR.2016.90.
4. Efron, B. Bootstrap methods: Another look at the jackknife. Ann. Stat. 1979, 7, 1–26. https://doi.org/10.1214/aos/1176344552.
5. Field, C.A.; Welsh, A.H. Bootstrapping clustered data. J. R. Stat. Soc. Ser. B Stat. Methodol. 2007, 69, 369–390. https://doi.org/10.1111/j.1467-9868.2007.00593.x.

https://doi.org/10.3390/jimaging12100487
